# Observation of the Assembly of the Nascent Mineral Core at the Nucleation Site of Human Mitochondrial Ferritin

**DOI:** 10.1021/jacs.5c01337

**Published:** 2025-04-14

**Authors:** Justin M. Bradley, Zinnia Bugg, Geoffrey R. Moore, Andrew M. Hemmings, Nick E. Le Brun

**Affiliations:** † Centre for Molecular and Structural Biochemistry, School of Chemistry, Pharmacy and Pharmacology, University of East Anglia, Norwich Research Park, Norwich NR4 7TJ, U.K.; ‡ Centre for Molecular and Structural Biochemistry, School of Biological Sciences, University of East Anglia, Norwich Research Park, Norwich NR4 7TJ, U.K.; # International Research Center for Food and Health, College of Food Science and Technology, Shanghai Ocean University, Nanhui New City, Shanghai 201306, China

## Abstract

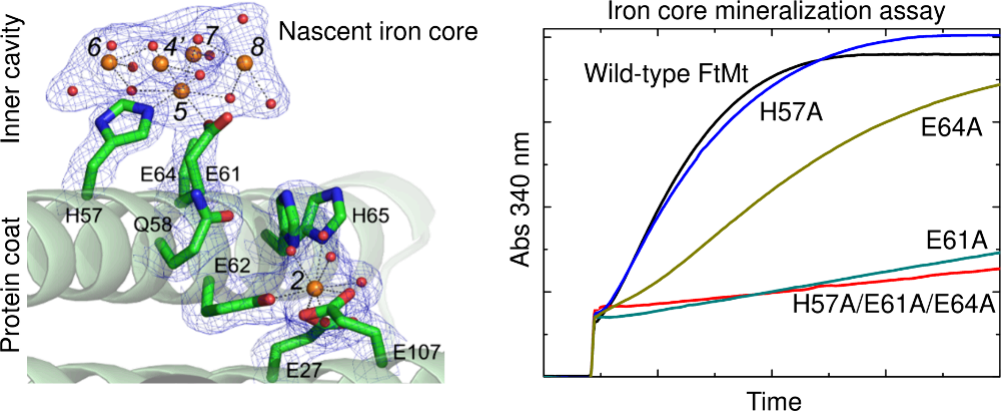

Ferritins play a crucial role in iron homeostasis and detoxification in organisms from all kingdoms of life. They are composed of 24 α-helical subunits arranged around an interior cavity where an iron-containing mineral core can be reversibly stored. Despite decades of study, leading to significant progress in defining the routes of Fe^2+^ uptake and the mechanism of its subsequent oxidation to Fe^3+^ at diiron catalytic sites termed ferroxidase centers, the process of core synthesis from the product of ferroxidase center activity remains poorly understood. In large part, this is due to the lack of high-resolution structural data on ferritin cores anchored to their nucleation sites on the inner surface of the protein. Mitochondrial ferritins are atypical of those found in higher eukaryotes in that they are homopolymers in which all subunits contain both a ferroxidase center and a presumed but undefined core nucleation site. Here, in conjunction with a novel method for producing iron-enriched ferritin crystals, we exploit these unusual features to structurally characterize both the nucleation site of mitochondrial ferritin and a pentanuclear, ferrihydrite-like iron-oxo cluster formed there. Kinetic data for wild-type and variant proteins confirmed the functional importance of this site, indicating a critical role for E61 in the transfer of Fe^3+^ from the ferroxidase center to the nascent mineral core.

## Introduction

Ferritins are multimeric proteins that perform important roles in iron homeostasis and oxidative stress response.^1−3^ These ubiquitous proteins are widely distributed across all domains of life. Examples isolated from animals^4^ and plants^5^ are exclusively 24mers of α-helical bundle subunits, while prokaryotes commonly express both 24meric examples^6^ and dodecameric ‘mini ferritins’.^7^ The sequence identity between different classes of 24meric ferritin can be as low as 20%^8^ but, despite this, the proteins exhibit remarkable similarity in structure. Structural models derived from X-ray diffraction data are available for all classes of cage-forming ferritins, and reveal that a four α-helical bundle motif constitutes the core of the monomeric unit of each.^9^ Furthermore, other than one example from the archaeon *Archaeoglobus fulgidus,*(10) all 24meric ferritins form cages of 432 symmetry, possessing three 4-fold, four 3-fold and six 2-fold symmetry axes. This results in a rhombic dodecahedral protein cage of 120 Å external diameter surrounding an approximately spherical interior cavity with a diameter of 80 Å. Channels at the 4-fold, 3-fold and, in prokaryotic proteins, 2-fold symmetry axes connect the interior cavity of the proteins to bulk solution.

Ferritin activity results in the oxidation of Fe^2+^ to Fe^3+^, and the formation of a hydrated ferric-oxy mineral stored within the central cavity of the protein cage. The ferroxidase center (FoC), an intrasubunit di-iron site, catalyzes the oxidation of two Fe^2+^ ions coupled to the two-electron reduction of either molecular oxygen or peroxide.^11^ Most animal ferritins are heteropolymers composed of H-chain subunits, which contain the FoC, and L-chain subunits that are isostructural but lack this catalytic site. L-chains do, however, contain a nucleation site on their inner surface^12^ that promotes mineralization. The relative expression levels of H- and L- chains are tissue dependent and dictate the composition of the heteropolymer, and thus modulate the iron oxidation and mineralization properties of the protein.^13^ In contrast, the ferritins of prokaryotes and plants exclusively contain H-chain-type subunits, each containing a FoC. However, the genomes of these organisms typically encode multiple ferritins^8^ with specialized cellular roles for which their iron oxidation and mineralization kinetics are optimized.^14^

In 2001 a gene encoding a new class of animal ferritin with an N-terminal mitochondrial targeting sequence was discovered, located in humans on chromosome 5q23.1.^15^ The product of this gene, which is 77% identical to human H-chain ferritin (HuHF, Figure S1), is targeted to the mitochondrial matrix, where it is proposed to play a role in oxidative stress management.^16^ It is unusual among animal ferritins in that it is a homopolymer, requiring that both the FoC and the nucleation site for mineral core formation be located on the same subunit type. Expression levels of this mitochondrial ferritin (FtMt) correlate with mitochondrial density rather than iron status,^17^ meaning detection of the protein is restricted to cells with high metabolic activity. Despite this limited distribution, FtMt has attracted much interest because misregulation is associated with a number of disease states, including neurodegeneration.^18^ Since the initial report of human FtMt, genes encoding putative mitochondrial ferritins have been identified in many animals such as primates, equidae, aves and cetacea.^19^

In vitro characterization of ferritins has resulted in significant progress in understanding the mechanism of Fe^2+^ acquisition and oxidation,^3,8^ but the process of mineral core formation remains poorly understood, due in large part to the lack of high resolution structural data. At present the protein data bank contains over 1300 ferritin structures refined to greater than 2.5 Å resolution with over 90% of these derived from X-ray diffraction data. However, in only two cases^20,21^ do the models derived from the data provide atomic level detail of multinuclear iron clusters anchored to the inner surface of the protein cage, and in neither case do these species result from FoC-catalyzed oxidation of Fe^2+^. Aerobic exposure of crystals of human L-chain ferritin (HuLF) to Mohr’s salt (ferrous ammonium sulfate) led to the accumulation of tri-iron clusters anchored to the protein at a putative mineral core nucleation site composed of three conserved glutamate residues E60, E61 and E64.^21^ However, the composition of this cluster, (μ^3^-oxo)Tris[(μ^2^-peroxo)(μ^2^-glutamato-κO: κO’)](glutamato-κO)(diaquo) tri-iron(III), is not consistent with spectroscopic and magnetic susceptibility data on ferritin cores. These studies suggest that the interior of ferritin cores are composed of a ferrihydrite-like mineral with a magnetite like material at the surface.^22^ Soaking of HuLF or horse spleen ferritin (90% L-chain) crystals with Fe^3+^ produced extended clusters based on 2 (μ^3^-oxo)Tris(μ^2^-glutamato-κO: κO’)tri-iron(III) motifs with 2 additional Fe^3+^ ions bridged by E140.^20^ While the absence of peroxide from these clusters better represents the composition of ferritin cores, they were not produced by protein-catalyzed oxidation of Fe^2+^ and therefore do not shed light on how this process occurs. Very recently, a cryo-transmission electron microscopy structure of *Streptomyces coelicolor* bacterioferritin (Bfr) revealed mineral linked to the protein at the inner surface of the 4-fold channels, but at less than 3 Å resolution offered no information on the chemical composition of the core.^23^

**Figure 1:**
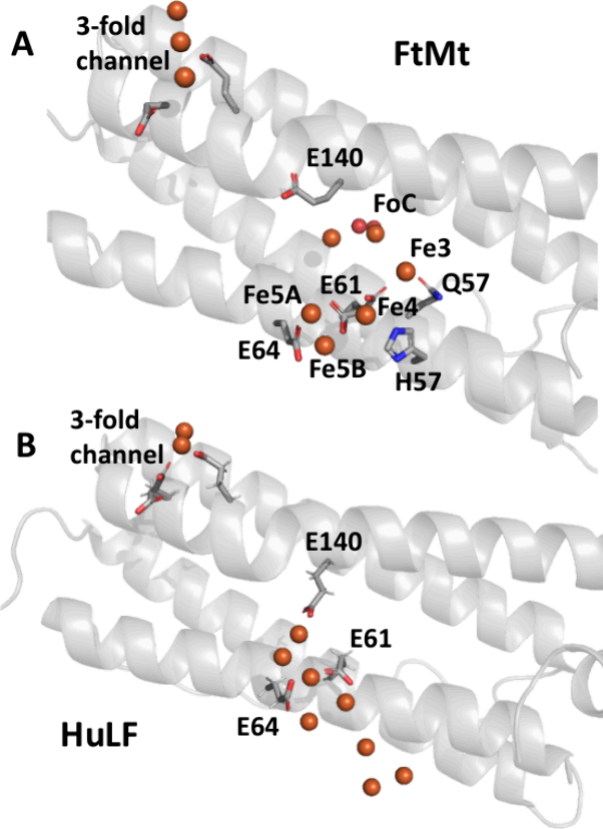
Iron transport and mineralization in ferritins. (A) Fe^2+^ enters animal ferritin via the 3-fold channel, and is proposed to be shuttled to the FoC via transient binding at a series of sites (Fe3–Fe5) on the inner surface of the protein. (B) The Glu residues involved in Fe^2+^ coordination at these sites are structurally equivalent to Fe^3+^-coordinating residues in the ferric-oxo cluster anchored to the inner surface of HuLF. Images generated from PDBs 7o68 (FtMt) and 6tsf (HuLF). Irons and oxygens are shown as orange/brown and red spheres, respectively.

In contrast to their bacterial counterparts, obtaining high resolution structural data on iron-enriched animal ferritins has proved challenging. Crystallization of the animal ferritins requires high pH, limiting the solubility of Fe^2+^ and thermodynamically favoring its oxidation to insoluble Fe^3+^ salts^24^ and also requires high concentrations of Mg^2+^, a competitive inhibitor of Fe^2+^ binding to ferritins.^25^ Consequently, the first iron-enriched structure of an animal ferritin was not reported until 2012^26^ and no high-resolution data was available until the development of a solid to solid diffusion method of iron enrichment in 2015.^25,27^ This technique was exploited to produce time-resolved, high-resolution structures of HuHF,^27^ the H’-subunit of *Rana catesbeiana*(25) (also known as frog M or H’) and FtMt.^28^ While these studies did not identify nucleation sites for mineral core formation or elucidate the structure of protein-associated Fe^3+^-containing mineral, they did identify a series of putative transient binding sites termed Fe3, Fe4 and, in FtMt only, Fe5. These transient binding sites were proposed to constitute waypoints in the transport of Fe^2+^ substrate from the inner surface of the 3-fold channel, the route of Fe^2+^ entry to HuHF and frog H’ and presumably to FtMt, to the FoC where it is oxidized to Fe^3+^ product^28^ (Figure 1).

Curiously these transient binding sites involve coordination of Fe^2+^ by glutamate residues (E61 in HuHF, E57 and E136 in frog H’ or E61, E64 and E140 in FtMt) structurally equivalent to those identified as constituting the nucleation site on HuLF. This, together with the observation that in *S. coelicolor* Bfr the mineral core nucleates at the 4-fold channel^23^ and the inconsistency of the iron-oxo species observed in HuLF structures with other data on ferritin cores, raises the question whether these structures truly represent the native nucleation site for iron biomineralization within human cytosolic ferritin, or simply the binding of adventitiously oxidized iron to a negatively charged area of the protein inner surface due to charge complementarity. Conversely, the transient binding sites identified in the H-chain proteins may not represent discrete waypoints in the transport of Fe^2+^ to the FoC; rather, the identified Glu residues may simply provide a negative electrostatic potential that accelerates incoming substrate toward its site of oxidation.

Here we address these questions, exploiting a novel method for time-resolved X-ray crystallography of the Fe^2+^ oxidation/mineralization process. Co-crystals of FtMt with Fe^2+^ and Mg^2+^ were grown anaerobically. Protein-catalyzed Fe^2+^ oxidation reactivity was then initiated by transferring crystals to aerobic well solutions, such that the rate of the initial in crystallo reaction was not limited by that of Fe^2+^ binding.

X-ray diffraction studies performed following extended exposure of crystals to O_2_ revealed a large volume of scattering density in the vicinity of the previously observed Fe5 site, which is identified as an iron-(hydr)oxo cluster forming at a core nucleation site. The functional relevance of this site was investigated by solution kinetic studies of variant proteins with iron-coordinating residues replaced by Ala. The data demonstrate that the identified nucleation site plays a key functional role in iron mineralization, and that movement of Fe^3+^ ions out of the FoC is inhibited in its absence. While no evidence for iron binding in the 3-fold channel was derived from structural data, solution kinetic studies confirmed the 3-fold channel as the route of iron entry into FtMt.

## Results

### Co-crystallization with Fe^2+^/Mg^2+^ Yielded Iron-Bound Forms of FtMt

Crystals of FtMt containing Fe^2+^ and Mg^2+^, grown and harvested anaerobically, diffracted to a resolution of 1.84 Å. The protein model deduced from these data overlaid well with previously reported structures, giving an overall RMSD for main chain atoms of 0.19 Å (160 residues) with that of the first reported magnesium-containing structure, and 0.18 Å (158 residues) with that following 5 min of anaerobic exposure to ferrous ammonium sulfate, pdb entries 1R03 and 7O6A, respectively.^28,29^ We therefore conclude that the anaerobic cocrystallization employed here had no significant effect on protein structure relative to conventional metal ion soaking of aerobically grown crystals.

**Figure 2:**
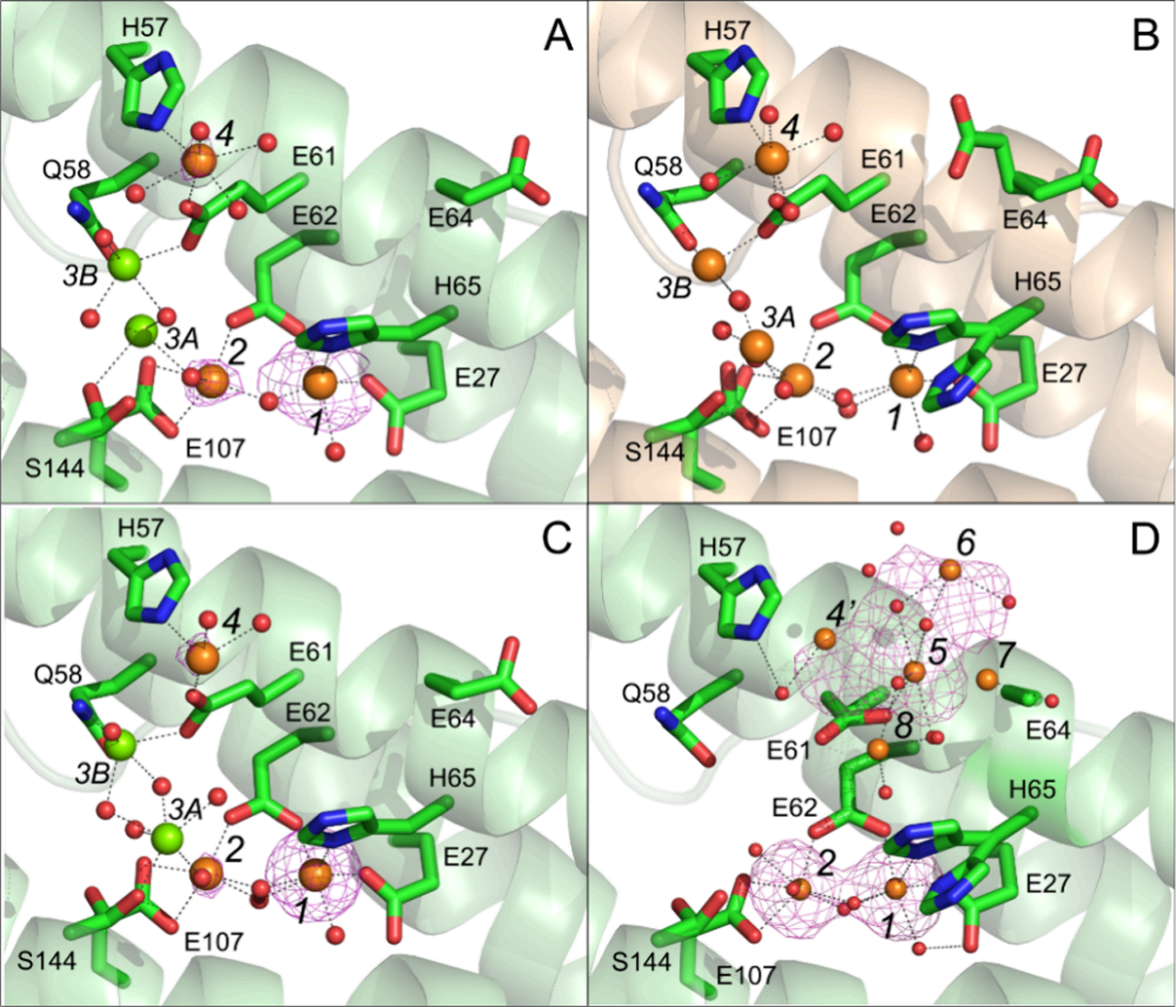
Exposure to O_2_ leads to formation of site of mineral core nucleation in iron-loaded FtMt. (A) Hydrated iron bound to site 4, a proposed transfer site^28^ involved in transport of Fe^2+^ substrate to the ferroxidase center in anaerobically harvested FtMt/Fe^2+^ cocrystals. The magenta mesh in this and panel (C) shows the anomalous difference Fourier map contoured at 6 σ. Metal binding sites are indicated by italicized black numbers. These include the locations of the proposed iron transfer sites 3A and 3B leading to the ferroxidase center^28^ occupied by magnesium ions in this structure. Iron positions are indicated by orange spheres, magnesium ions by green spheres, oxygen by red spheres. Metal coordination bonds are shown as black dashed lines. Individual residues coordinating the metal ions are shown in stick format and labeled. In this panel and panel (C) the minor conformer (28% occupancy) of His65 is not shown. (B) The equivalent structure derived from crystals anaerobically exposed to ferrous ammonium sulfate for 5 min (PDB entry 7O6A). (C) As (A) but for cocrystals exposed to aerobic well solution for 2 min prior to harvesting showing iron occupancies in sites 2 and 4 have decreased and peroxide is bound at the ferroxidase center. (D) The appearance of a large volume of anomalous difference electron density, modeled as an iron-oxo cluster in the vicinity of site 5^28^ following exposure of cocrystals to aerobic well solution for 20 min prior to harvesting. Three iron sites (4’, 5 and 6) were found at peak heights at or above 4.0 σ in the anomalous difference electron density map (contoured at 4 σ in this panel). Site 4’ lies 1.8 Å from site 4 found in the structure of anaerobically harvested FtMt/Fe^2+^. Iron sites labeled 7 and 8 were located by MR-SAD^30^ and confirmed by ANODE.^31^ These are found at peak heights of 3.2 σ and 3.0 σ, respectively. Note that the side chain of residue E64 is presumed disordered as no significant electron density beyond atom Cγ was observed. In this panel a second conformer of His65 with significant occupancy (53%) is also shown.

Iron and magnesium ion binding sites were identified as described in the Methods section. This process revealed peaks in the anomalous scattering data corresponding to sites Fe1 and Fe2 at the FoC, and Fe4 close by on the inner surface, as reported by Ciambellotti et al.,^28^Figure 2A and 1B, Figure S2. Fe2 at the FoC was at lower occupancy than Fe1 (Table 1) with an Fe–Fe distance of 3.37 Å (Table 2) and bridging oxygen that is very likely a water. This form of the FoC corresponds to the di-Fe^2+^ form, with an Fe–Fe distance very similar to that previously reported for anaerobic crystals exposed to ferrous ammonium sulfate.^28^ Further details of the di-Fe^2+^ FoC are given in Figure S2 and Table 2 and Table S3. Site Fe4 is coordinated by H57 and E61 (Figure 2A) and was previously concluded to be a transient site for Fe^2+^en route from the 3-fold channels to the FoC.^28^ No evidence for iron coordinated at other putative transient binding sites (Fe3A/B or Fe5), or within the 3-fold channels themselves, was found. Instead, Mg^2+^ ions were found at these positions, Figure 2A and Figure S3. Iron was, however, found at a site within the 4-fold channels, Figure S4, as reported previously^28^ and fractional occupancies of the FoC and Fe4 sites were remarkably similar to the previous report.

**Table 1:** Occupancies of the Iron-Binding Sites in the Refined Structures

Fe Site	1	2	3	4	5	6	7	8	4-fold
0 min	0.86	0.40	-	0.48	-	-	-	-	1.00
2 min	0.97	0.26	-	0.29	-	-	-	-	1.00
20 min	0.61	0.64	-	0.72	0.59	0.62	0.78	0.63	1.00
AAA^a^	1.00	0.73	-	-	-	-	-	-	0.99
HuHF^b^	1.00	0.91	-	0.52	-	-	-	-	0.85

a H57A/E61A/E64A FtMt triple variant 20 min O_2_ soak.

b Human H-chain ferritin 20 min O_2_ soak.

**Table 2:** Geometries of the Ferroxidase Centers in the Refined Structures^a^^,^^b^

	r/Å (Fe–Fe)	θ_1_/° (Fe–O1–Fe)	r/A (O1–O2)	θ_2_/° (Fe–O2–Fe)
0 min	3.37 ± 0.19	139.0	-	-
2 min	3.51 ± 0.08	127.8	1.40	120.0
20 min	3.10 ± 0.12	94.7	2.60	94.1
AAA^c^	3.44 ± 0.06	113.6	2.40	92.6
HuHF^d^	3.47 ± 0.12	113.3	2.35	93.7

a Oxygen atoms O1, O2 bridge the iron sites 1 and 2 at the ferroxidase center.

b Estimated errors in ferroxidase center iron positions were calculated according to the method of Kumar et al.^33^

c H57A/E61A/E64A FtMt triple variant 20 min O_2_ soak.

d Human H-chain ferritin 20 min O_2_ soak.

Figure 2B shows a similar view of the area in the vicinity of the FoC generated from the previously reported structure of FtMt (pdb entry 7O6A),^28^ demonstrating that the iron-coordinating residues adopt identical conformations in both structures. However, while H65 in the model reported here has greater *B* factors than the other FoC ligands, the electron density does not support an alternate conformation, as reported in 7O6A.

### E61 and E64 Constitute an Inner Surface Nascent Iron Mineral Binding Site in FtMt

Co-crystallization of FtMt with Fe^2+^ allowed the progress of the iron mineralization reaction to be studied by freezing crystals at defined time points following exposure to O_2_. Crystals frozen following soaking in O_2_-containing well solution for 2 min diffracted to a resolution of 1.48 Å. The main chain atoms of the model derived from these data overlaid those of the anaerobic structure above with RMSD of 0.13 Å (157 residues).

**Figure 3:**
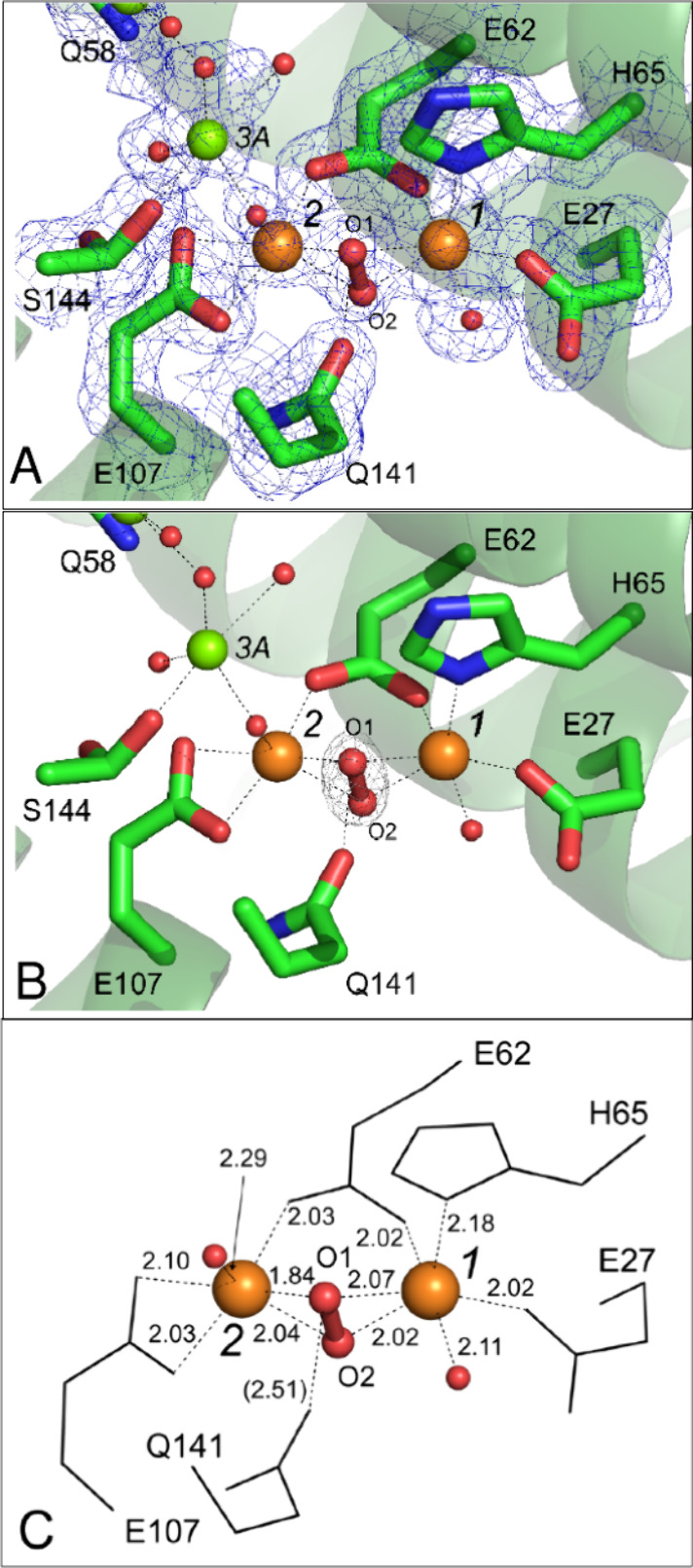
Ferroxidase center in FtMt/Fe^2+^ cocrystals exposed to aerobic well solution for 2 min prior to harvest. (A) Blue mesh shows the Sigma-A weighted Fourier (2mFo-DFc) map contoured at 1.1 σ. Iron positions are indicated by orange spheres, oxygen by red spheres. Individual residues are shown in stick format and labeled. Metal-binding sites are indicated by italicized black numbers and metal coordination bonds are shown as black dashed lines. The oxygen atoms of the peroxide bridging the ferroxidase iron positions are labeled O1 and O2. (B) as (A) but gray mesh shows the peroxide omit map contoured at 6σ. (C) as (A) but showing detail of the geometry of the FoC. Individual residues are shown in wire format and labeled. The lengths of metal coordination bonds are shown in Ångstrom units. The length of the hydrogen bond from the side chain of Gln141 to peroxide atom O1 is indicated in brackets. Other geometry values are provided in Table 2.

The structure in the region of the FoC is shown in Figure 2C. The positions of the residues acting as ligands to the FoC were unchanged relative to the anaerobic structure. Metal ion occupancy at Fe1 of the FoC was somewhat higher, while that of Fe2 was decreased (Table 1). While there was a small increase in the separation between Fe1 and Fe2 from 3.37 to 3.51 Å (Table 2), this was not significant with respect to the estimated error in atomic coordinates. Strikingly, peroxide, the product of O_2_ reduction that is coupled to Fe^2+^ oxidation at the FoC^19,32^ was coordinated side on between the two metals (Fe–Fe μ-η^2^:η^2^ side-on coordination), Figure 3, in a similar fashion to that previously reported for crystals of FtMt aerobically exposed to ferrous ammonium sulfate for 3–5 min.^28^ This form of the FoC could correspond to a peroxide-bound di-Fe^3+^ site, or peroxide bound to a mixed valent form of the FoC that we recently showed forms in solution under low O_2_ conditions.^19^ Occupancy of Fe4 was decreased following 2 min exposure to O_2_ (Table 1), and sites Fe3*A*/3B remained occupied by Mg^2+^ ions. The only other peak in the Bijvoet-difference Fourier map corresponded to iron bound in the 4-fold channels (Figure S4).

Changes of greater significance were observed on increasing the length of O_2_ exposure to 20 min prior to freezing. Crystals diffracted to 1.97 Å resolution and the model derived from these data overlaid that of the anaerobic structure with overall RMSD for main chain atoms of 0.19 Å (139 residues), indicating no change in the fold of the protein on prolonged O_2_ exposure, the major differences being in the unstructured region at the N terminus of the peptide. The separation between Fe1 and Fe2 was decreased to 3.10 Å, and the occupancies of the two sites were equal at ∼ 0.6 (Tables 1 and 2). Two bridging O atoms were retained, but with an O–O distance too long for a bonding interaction, indicating that peroxide was lost and replaced by oxide and/or hydroxide ions (Table 2, Figure S5), along with oxidation of Fe^2+^ at the FoC generating the di-Fe^3+^ form of the center.

**Figure 4:**
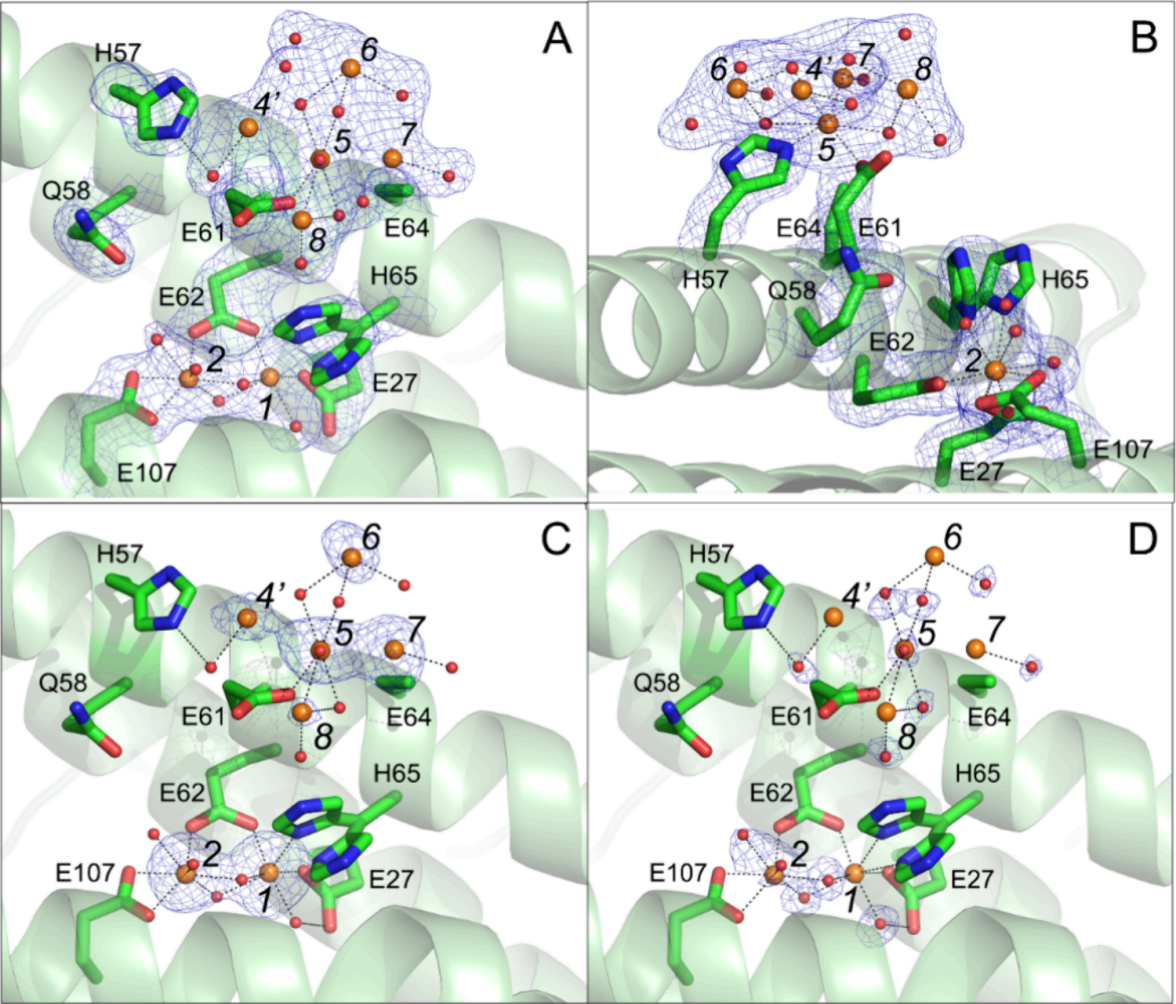
An iron-oxo cluster on the inner surface of FtMt. (A, B) Approximately orthogonal views of an iron-oxo cluster in the vicinity of site 5^28^ following exposure of cocrystals to aerobic well solution for 20 min prior to harvesting. Blue mesh shows the Sigma-A weighted Fourier (2mFo-DFc) map contoured at 1.3 σ within 4 Å of potential iron metal coordinating residues and the metal ions themselves. Iron positions are indicated by orange spheres, oxygen by red spheres. Individual residues are shown in stick format and labeled. Note that the side chain of residue E64 is presumed disordered as no significant electron density beyond atom Cγ was observed. Metal coordination bonds are shown as black dashed lines. (C), (D) As (A) but blue mesh shows the iron omit and oxygen omit maps, respectively, contoured at 3σ.

**Figure 5:**
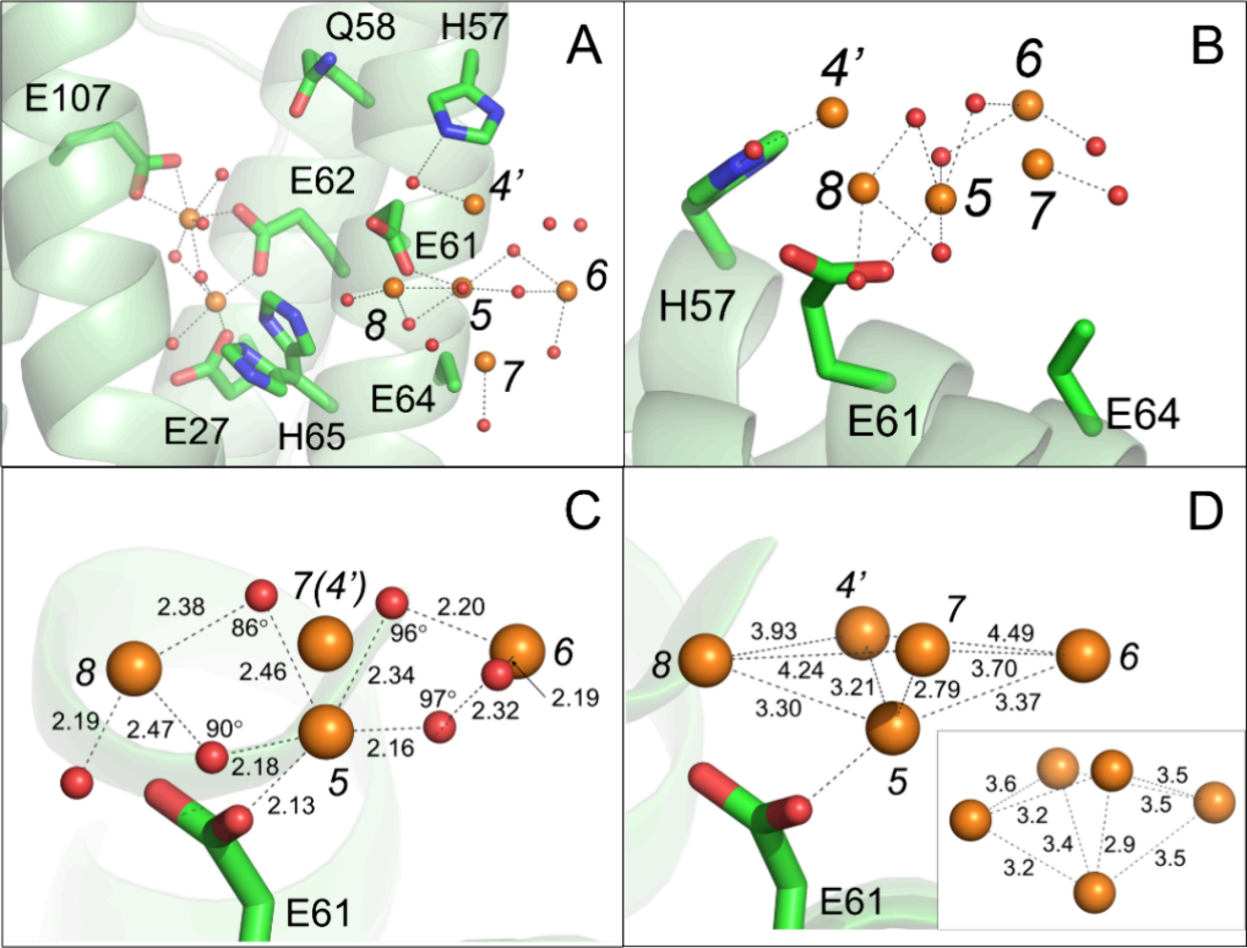
Iron-oxo cluster. (A) A view from inside the ferritin protein cage showing the ferroxidase center and iron-oxo cluster in the structure of FtMt observed following exposure of cocrystals to aerobic well solution for 20 min prior to harvesting. Iron positions are indicated by orange spheres, oxygen by red spheres. Individual residues coordinating the metal ions are shown in stick format and labeled. Metal binding sites are indicated by italicized black numbers and metal coordination bonds are shown as black dashed lines. Cartoon representation of FtMt polypeptide shown in green. (B) as (A) but showing an orthogonal close-up view of the iron-oxo cluster. (C) The geometry of the nascent mineral core. Interatomic distances where shown are in Ångstrom, angles in degrees. Note that in this view Fe4’ is occluded by Fe7. (D) Geometry of the cluster iron substructure. A putative platform for mineral core growth provided by iron sites 4’, 6, 7, and 8. Inset shows a comparable arrangement found within a proposed structure for ferrihydrite.^34^

At the inner surface, residue E61 adopted a conformation in which the side chain is directed toward E64, and the Bijvoet-difference Fourier map contained a significant peak between the two carboxylates. This corresponded to the previously reported Fe5 site, interpreted as a transient site for iron uptake into the FoC.^28^ However, in our data a large area of electron density was observed to extend into the interior cavity from this site. This was modeled as four further iron ions (Figure 2D and Figure 4), with occupancies of all 5 irons (Fe4’ – Fe8) in the range 0.6 – 0.8, Table 1. Bridging and terminal oxygens of the iron-oxo cluster were also identified. Thus, the 20 min exposure to O_2_ revealed the presence of an inner surface site, involving residues H57, E61 and E64, capable of binding an iron-oxo cluster containing at least five iron ions, see Figure 4 and Figure 5 for further details.

Site Fe4’ sits 1.8 Å from site Fe4 identified in the structure following 2 min of O_2_ exposure, an interatomic separation too short for both iron sites to be occupied simultaneously. Growth of the iron-oxo cluster on the inner surface of FtMt therefore occludes the putative transient binding site Fe4, preventing the binding of incoming Fe^2+^ substrate here. Control data collected on crystals of HuHF grown under identical conditions and treated equivalently demonstrated that the formation of an iron-oxo cluster is unique to FtMt and not an artifact of the cocrystallization method, Figure S6. Crystals of a H57A/E61A/E64A triple variant of FtMt, designed to eliminate the iron-oxo cluster binding site, also showed no evidence of either unmodeled density or anomalous scattering in the vicinity of the Fe5 site (Figure S6). Other than the side chains of the substituted residues, the structures derived from crystals of the variant protein were identical to those from equivalently treated crystals of wild-type FtMt, the structures overlaying with overall RMSD for main chain atoms of 0.110 Å (150 residues).

### The Geometry of the Nascent Mineral Core

The nascent mineral core is made up of a network of three iron sites (Fe5, Fe6 and Fe8) bounded on either side by two peripheral sites (Fe4’ and Fe7), see Figure 5A-C. The sites Fe4’, Fe6, Fe7 and Fe8 form a surface facing the ferritin inner cavity, being made up of two roughly congruent triangles sharing a common base formed by sites Fe4’, Fe6 and Fe7, and Fe4’, Fe8 and Fe7. This platform may serve as a foundation to promote further growth of the mineral.

Interestingly, the arrangement of iron sites of the cluster in the refined structure so described bears resemblance to a distorted version of that previously proposed for ferrihydrite^34^ wherein sites 5, 4’ 6 and 8 would correspond to octahedral sites and 7 to a tetrahedral site (Figure 5D). We note, however, that the crystal structure observed represents only an average, in this case symmetry-averaged over the 24 copies of the FtMt protein subunit, and a degree of spatial disorder is likely. This is exacerbated in time-resolved studies where O_2_ diffuses into the crystal and turnover at the FoC occurs to generate the nascent mineral core. For this reason, we have restricted our description of the mineral core to those sites corresponding to the strongest peaks in the difference and anomalous difference Fourier maps.

### E61 and E64 Constitute the Nucleation Site of the FtMt Mineral Core

Iron sites in the vicinity of residues H57, E61 and E64 were previously observed following iron enrichment of FtMt crystals.^28^ These were interpreted as transient binding sites involved in the translocation of incoming Fe^2+^ substrate from the 3-fold channel to the FoC.^28^ However, the iron-oxo cluster observed here suggests that site Fe5 is the site of mineral core nucleation rather than an additional transient binding site for incoming substrate. The rate of iron oxidation at the FoC, established by monitoring the transient absorbance at 650 nm due to the formation of a characteristic diferric-peroxo intermediate, is known to be limited by iron binding^19^ and is kinetically distinct from that of mineral formation. Absorbance-monitored solution activity assays were therefore employed to elucidate the functional significance of the Fe5 site for both transport of Fe^2+^ to the FoC and mineralization of Fe^3+^.

**Figure 6:**
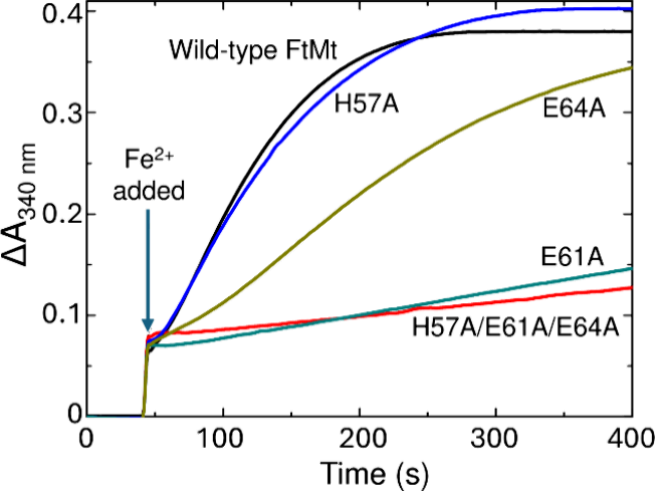
Iron mineralization by FtMt and inner surface variants. The increase in absorbance at 340 nm as a function of time following the aerobic addition of Fe^2+^ to a final concentration of 200 μm to 0.5 μm solutions of FtMt. The arrow indicates the point of Fe^2+^ addition.

Ligand to metal charge transfer transitions of ferric-oxo species give rise to broad absorbance features centered at 340 nm, with extinction coefficients of approximately 2000 m^–1^ cm^–1^ at this wavelength. Rates of Fe^2+^ oxidation can therefore be deduced from the rate of increase in 340 nm absorbance following aerobic mixing with ferritin. Figure 6 shows the increase in absorbance at 340 nm following the aerobic addition of 400 equiv of Fe^2+^ to wild-type, single variants H57A, E61A, and E64A FtMt, and triple variant H57A/E61A/E64A FtMt. Initial velocities of the mineralization reaction can be calculated from the slope of the linear region of the plots before substrate availability becomes rate-limiting (Table 3). Data for the triple variant showed that deletion of the H57/E61/E64 inner surface sites severely impacts the ability of the protein to form a mineral core.

**Table 3:** Kinetic Parameters for Fe^2+^ Oxidation by FtMt and Variants

	Protein							
	Wild-type FtMt	H57A/E61A/E64A	H57A	E61A	E64A	D131A^c^	E134A	E140A
Mineralization rate (μm min^–1^)^a^	79	3.9	71	6.9	24	26	80	29
Rate constant Fe^2+^ binding (10^5^m^–1^ s^–1^)^b^	3.0	0.93	4.3	1.3	1.7	-	1.7	0.76

a Determined from the initial velocity of the iron mineralization reaction.

b Calculated second-order rate constant for Fe^2+^ binding to the FoC of each of the proteins studied.

c No rate constant for Fe^2+^ binding to the FoC is available for variant D131A as this protein has no rapid phase of oxidation for which iron binding is rate-limiting.

An indication of the individual contributions of the three residues was provided by data for the single variants. These demonstrated that E61 is the critical residue for core nucleation, with substitution of this residue alone having an almost identical impact to substitution of all three potentially coordinating residues. In contrast, substitution of H57 had no significant effect other than the introduction of a brief lag phase prior to mineralization. This lag phase could be eliminated by preincubation of the protein with ≥ 72 equiv of Fe^2+^ prior to initiating the mineralization assay (Figure S7). The activity of variant E64A was intermediate between that of wild-type and variant E61A, suggesting a role for E64 in binding Fe^3+^ at the nucleation site, but one that is less critical for mineralization than that of E61.

Ferritins such as HuLF that lack a FoC exhibit much lower mineralization activity than the H-chain and H-chain-like proteins.^35^ Therefore, the impaired mineralization observed for variants E61A, E64A and H57A/E61A/E64A could be the result of loss of FoC activity. Under the assay conditions employed, FoC turnover typically occurs on the time scale of 100 ms to several seconds, while mineral core formation requires several minutes. Consequently, FoC activity of the variant proteins was compared to that of wild-type protein by using the absorbance at 340 nm to monitor reactivity for 10 s following stopped-flow mixing of aerobic solutions of protein and Fe^2+^. The resulting data are shown in Figure S8, along with the Fe^2+^ concentration dependence of the apparent first-order rate constant for rapid oxidation extracted by fitting the data to a biexponential function. Biphasic kinetics were only observed at iron loadings >48 Fe/cage, sufficient to initiate the process of mineralization, with apparent first-order rate constants of ≤ 0.1 s^–1^ dependent on the FtMt variant used. In contrast FoC-catalyzed oxidation of Fe^2+^ binding to apo sites occurred with apparent first-order rate constants of between 2 and 20 s^–1^, depending on the variant and Fe^2+^ concentration employed, meaning the two processes are readily deconvoluted from one another. The data demonstrate that the FoC remained competent for Fe^2+^ oxidation in all variants, and that Fe^2+^ binding is rate-limiting for the rapid phase of turnover. Table 3 lists the second-order rate constant for Fe^2+^ binding to the FoC for wild-type FtMt and each of the variant proteins. Substitution of the neutral H57 had a negligible effect on the rate of the FoC reaction, while that of substitution of E61 was greater than E64 and appeared to be cumulative in variant H57A/E61A/E64A.

### Nucleation Site Residue E61 is Critical to Release of Fe^3+^ from the FoC

Stopped-flow absorbance demonstrated that the FoC remains functional following substitution of any of the ligands at the inner surface nucleation site, and that the rate of FoC-catalyzed Fe^2+^ oxidation is too great to be limiting for mineral core formation. Thus, the rate-limiting step of iron mineralization is likely the release of oxidized product to regenerate vacant FoC sites. Since the rapid phase of Fe^2+^ oxidation requires vacant FoC sites, the rate at which this activity is regenerated following incubation of ferritin with iron can be used to probe the rate of product release.^36^

**Figure 7:**
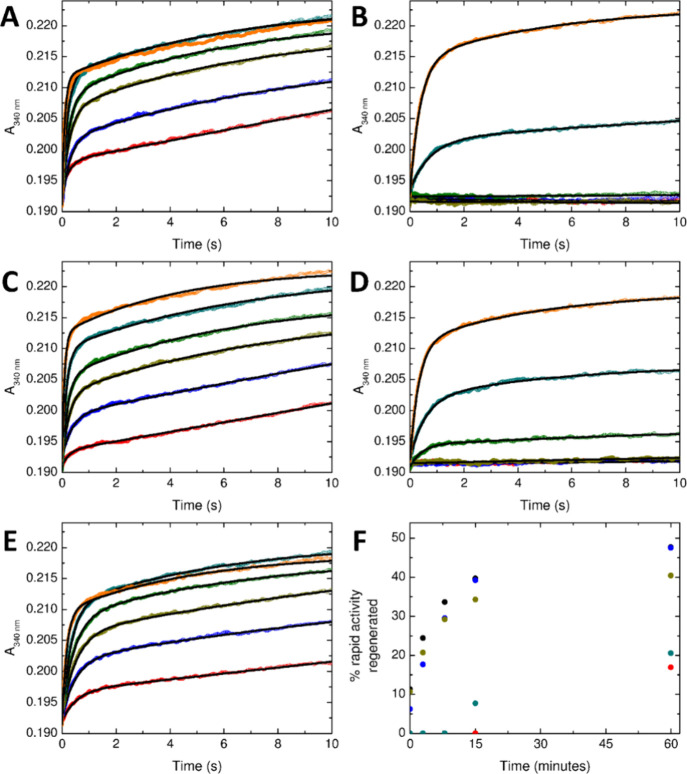
Regeneration of rapid Fe^2+^ oxidation by FtMt. The increase in absorbance at 340 nm following the aerobic mixing of Fe^2+^ and FtMt either immediately following the oxidation of 200 equiv of Fe^2+^ (red) or after a further period of 3 (blue), 8 (yellow), 15 (green), 60 (cyan) or 1000 (orange) min. Data for (A) wild-type, (B) H57A/E61A/E64A, (C) H57A, (D) E61A or (E) E64A FtMt; fits to a biexponential decay are shown as black lines. Panel (F) shows the percentage of the rapid activity of the corresponding apo protein regenerated for time points up to 60 min for wild-type (black) or variant H57A/E61A/E64A (red), H57A (blue), E61A (dark cyan) or E64A (dark yellow) FtMt.

Wild-type and variant FtMt was aerobically mixed with 200 equiv of Fe^2+^ and oxidation allowed to proceed to completion. Figure 7 shows increases in 340 nm absorbance following the stopped-flow mixing of wild-type, H57A, E61A, E64A or H57A/E61A/E64A proteins containing 200 equiv of Fe^3+^ with a further 72 equiv of Fe^2+^ at various time points after completion of the initial oxidation reaction.

The data demonstrate that, in the absence of further incoming Fe^2+^ substrate, the rate of product release in variants H57A and E64A, as judged by the extent to which rapid FoC activity recovered, was indistinguishable from the wild-type protein. However, variants E61A and H57A/E61A/E64A were both inhibited to the same extent. Therefore, of the 3 residues acting as ligands to the observed iron-oxo cluster, E61 is critical to release of Fe^3+^ from the FoC of FtMt. Table S4 lists the parameters describing rapid Fe^2+^ oxidation extracted from the biexponential fits shown in Figure 7.

### Fe^2+^ Substrate Enters FtMt via the 3-fold Channel

The 3-fold channels have been demonstrated to be the entry point for Fe^2+^ into the cytosolic animal ferritins.^2,37−39^ The high degree of sequence identity between mitochondrial and cytosolic H-chain ferritins extends to the region of the 3-fold channel (Figure S9). Therefore, this channel is expected to constitute the entry point for Fe^2+^ substrate for the mitochondrial ferritins also. Indeed, iron bound in the 3-fold channel was observed in previously reported structures of FtMt and the presence of iron bound to the inner surface was interpreted as revealing transient binding sites en route from the 3-fold channel to the FoC.^28^ However, our structural data revealed no evidence of iron binding in the 3-fold channel, or at any point on the inner surface of the protein, other than directly beneath the FoC (at sites Fe4 – Fe8, see above).

The only other areas of electron density associated with anomalous scattering were located within the 4-fold channel and a minor site on the exterior face of the protein in the vicinity of D84. Therefore, FtMt variants D131A, E134A and E140A were constructed to investigate the effect of substituting residues equivalent to those previously demonstrated to be important for iron uptake in frog H’ ferritin.^37,39^ D131 and E134 are located within the 3-fold channel, while E140 is located on the inner surface of the protein between the 3-fold channel and the FoC. As expected, given that iron uptake is not rate-limiting for core formation, the effect of each substitution on mineralization activity was minimal (Figure S10, Table 3), with D131A and E140A showing some effect on mineralization rate. In contrast, the D131A substitution inhibited uptake of Fe^2+^ to the extent that rapid iron oxidation at the FoC was abolished, while variants E134A and E140A had second-order rate constants for Fe^2+^ binding at the FoC that were ∼ 40% and ∼ 20% of the wild-type value, respectively (Figure S10, Table 3). This is precisely the pattern of inhibition of Fe^2+^ uptake reported for frog H’ ferritin following substitution of the equivalent residues by alanine.^37^ Thus, the 3-fold channel also constitutes the major route of iron entry for FtMt.

## Discussion

Previous reports of iron-bound structures of animal H-chain ferritins identified sites spanning the region between the 3-fold channel and the FoC. These were interpreted as transient binding sites for uptake of Fe^2+^ substrate.^25,27,28^ Our solution kinetic data are inconsistent with this interpretation. The consequences of disruption of the 3-fold channel by site-directed mutagenesis for the kinetics of Fe^2+^ uptake confirmed that this channel is the major route for Fe^2+^ entry into the protein, despite the observation of iron bound to the 4-fold channel in structural models derived from iron-enriched crystals. In contrast, the rate at which Fe^2+^ is transported to the FoC was unaffected by deletion of a site Fe4 ligand in variant H57A. However, replacing negatively charged Glu residues identified as ligands to these sites significantly impacted the rate of Fe^2+^ binding to the FoC. While this suggests that rates of Fe^2+^ binding are in most part determined by electrostatic interactions, our structural models revealed multiple conformations of the carboxylate side chains similar to those previously reported,^28^ suggestive of a role for their conformational flexibility.

**Figure 8:**
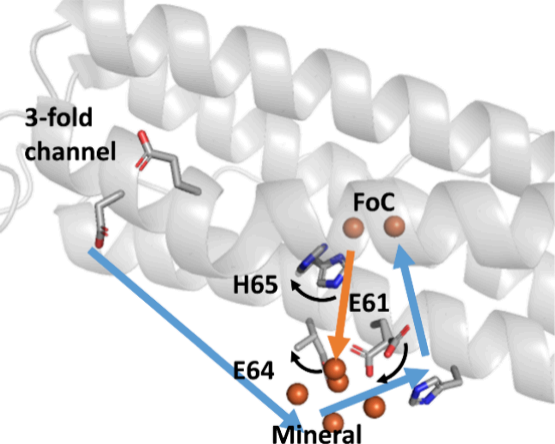
Coordinated side chain movements separate the Fe^2+^ uptake and Fe^3+^ release pathways in FtMt. Blue arrows indicate the route of Fe^2+^ entry into, and orange arrows the route of Fe^3+^ exit from, the FoC to the developing mineral core. Curved black arrows indicate the direction of side chain movement on Fe^3+^ release from the FoC. Movement of H65 triggers Fe^3+^ release from the FoC, with subsequent rearrangement of E61 to transport the product to site Fe5 on the inner surface for incorporation into the growing mineral core. Data following exposure of crystals to O_2_ for 20 min did not permit placement of the carboxylate group of E64 but the rotomer conformation is clearly different to that prior to cluster formation. We propose that this movement guides incoming Fe^2+^ away from the route of Fe^3+^ release and a breakdown in this separation is the origin of decreased mineralization activity in variant E64A.

Our novel method for iron enriching FtMt crystals resulted in iron loading of the protein prior to the initiation of reactivity. As a consequence, the rate of reaction was not limited by Fe^2+^ transport, resulting in the observation of a pentanuclear iron-containing cluster at site Fe5, anchored to the inner surface of the protein via E61 and E64. Assembly of this cluster occludes Fe^2+^ binding to site Fe4, further questioning the role of this site as a nonredundant waypoint for the uptake of substrate into the FoC. However, our solution kinetic data demonstrated a critical role for E61 in shuttling Fe^3+^ out of the FoC and delivering it into the nascent mineral core developing at site Fe5. In contrast, substitution of E64 did not affect stability of Fe^3+^ bound to the FoC but did inhibit the rate of mineralization in protein incubated with 400 equiv of Fe^2+^. Conformational flexibility in E64 and E61 may therefore allow these residues to act co-operatively to separate routes of Fe^2+^ uptake into the FoC and Fe^3+^ release from it. The change in conformation in E61 to deliver Fe^3+^ into the core may trigger rearrangement of E64, creating an electrostatic gradient that guides incoming Fe^2+^ away from the route of Fe^3+^ release. Thus, in variant E64A we predict that these two routes clash, inhibiting the rate of mineral core formation in similar fashion to that proposed for variant D65A of the prokaryotic ferritin *Syn*Ftn.^40^Figure 8 summaries this model for routes of Fe^2+^ entry into and Fe^3+^ release from the FoC, leading to mineralization.

The nascent mineral core observed in this study is anchored to the protein via the side chains of E61 and E64, structurally equivalent to two of the residues identified as ligands to the iron clusters on the inner surface of HuLF. This suggests that the nucleation sites of cytosolic and mitochondrial ferritins are highly related. The anchoring of the mineral core to the protein inner surface at the 4-fold channel of *S. coelicolor* Bfr^23^ would therefore represent a further example of diversity in structure and mechanism within the ferritin family.^8^

Finally, our data differ from that reported for HuLF in terms of the structure and composition of the iron-oxo cluster formed at the putative nucleation site. While the resolution of the data reported here only allowed confident placement of five iron ions, their topology resembles that proposed for the structure of ferrihydrite.^30^ Therefore, we propose that the mineral species generated as the result of FoC activity does indeed closely resemble ferrihydrite. The pentanuclear cluster reported here occludes site Fe4, preventing Fe^2+^ binding here. It therefore seems reasonable to postulate that, as the mineral core continues to grow, it will eventually prevent access of incoming Fe^2+^ substrate to FoCs, inhibiting the rate of its oxidation. Absorption of this unreacted substrate into the surface of the mineral core may generate the Fe(II)Fe(III)_2_O_4_ inverse spinel material resembling magnetite at the core surface reported in earlier studies.^22^

## Methods

### Protein Overproduction and Purification

Plasmids encoding FtMt, HuHF and variant proteins based on the pET21a expression vector were purchased from Genscript (Netherlands). All FtMt constructs encoded for proteins that lacked the predicted mitochondrial targeting sequence and first 9 amino acid residues at the N terminus of the mature protein such that the sequence identity of the products with the equivalent HuHF was ≥ 80%. Proteins were expressed from *Escherichia coli* strain BL21(DE3). Cells spread onto LB agar plates containing 100 μg mL^–1^ ampicillin were incubated overnight at 37 °C. All cultures in liquid media were grown at 37 °C, 200 rpm shaking unless otherwise stated and contained 100 μg mL^–1^ ampicillin. Single colonies were picked into 5 mL liquid media (LB) and grown on throughout the day. 400 μL of the resulting cell culture was used to inoculate 80 mL of LB that was grown to saturation overnight. 50 mL of the saturated culture was diluted 1 part in 100 into 5 L of LB and grown on until the optical density at 600 nm was in the range 0.6–0.8. Gene expression was induced by adding isopropyl β–d-1 thiogalactopyranoside (IPTG) to a final concentration of 100 μm. Cultures were grown on for a further 20 h at 30 °C, 90 rpm shaking prior to harvesting by centrifugation.

Pellets were resuspended in 20 mm HEPES, 100 mm KCl, 0.1 mm EDTA, pH 7.8 (buffer A) and the cells disrupted by sonication. Debris was removed by centrifugation at 40000 x *g*, 1 °C for 45 min. Thermally unstable proteins were precipitated from the supernatant by heating to 65 °C for 15 min and removed by a further round of centrifugation as above. Ferritin was precipitated from the supernatant by the addition of ammonium sulfate to a concentration of 0.55 g mL^–1^. Precipitated protein was pelleted by a further round of centrifugation before dissolving in a minimum volume of buffer A and dialyzing against 1 L of identical buffer for a minimum of 12 h. Contaminating proteins were removed by size exclusion chromatography (HiPrep 26/60 Sephacryl S300HR, Cytiva) and contaminating DNA by anion exchange chromatography (HiTrap Q FF, Cytiva). For the latter, protein solutions were loaded in buffer A and eluted by stepping to 30% buffer B (20 mm HEPES, 100 mm KCl, 1 m NaCl, 0.1 mm EDTA, pH 7.8).

Protein as isolated contained some iron that was removed using the method of Bauminger et al.^41^ Following iron removal, protein was exchanged into 100 mm MES pH 6.5 by centrifugation over a 10 kDa molecular weight cut off cellulose membrane (Millipore). The absence of contaminating proteins was confirmed using SDS-PAGE and the ferritins judged free from DNA contamination once the ratio of absorbance at 280 nm over 260 nm reached 1.5. Protein concentration was determined by absorbance assuming ε_280 nm_ = 4.08 × 10^5^m^–1^ cm^–1^ for the 24meric protein cage.^32^

### Protein Crystallization and Structure Solution

Wild-type FtMt (2 mg mL^–1^), HuHF (10 mg mL^–1^) or H57A/E61A/E64A FtMt (2.4 mg mL^–1^) exchanged into 20 mm MES pH 6.5 in 2 μL drops were mixed with an equal volume of well solution (0.1 m bicine, 2.0 m magnesium chloride, 100 mm sodium chloride, 60 mm ferrous chloride, 3 mm sodium azide, pH 9.0) in a nitrogen-filled chamber (Belle technology, [O_2_] < 10 ppm) and equilibrated in sitting drops by vapor diffusion against 200 μL of the same well solution. Crystals of bipyramidal morphology appeared within 24 h and grew to optimum size (100–150 μm) in approximately 1 week. Crystals were either soaked in well solution with ambient dissolved O_2_ concentration for 2 or 20 min, or in an equivalent O_2_-free solution within a nitrogen-filled chamber. Following this treatment, crystals were transferred to cryo-protectant comprising the well solution, with magnesium chloride concentration increased to 2.2 m, containing 30% (v/v) glycerol prior to flash freezing in liquid nitrogen.

Diffraction data was collected on beamlines i03 and i04 at the Diamond Light Source (Didcot, UK), using wavelength 0.9763 Å for high resolution data. Additional, highly redundant anomalous scattering data was collected from the same or identically treated crystals at wavelengths corresponding to the peak (1.7395 Å) and on the low energy side (1.7500 Å) of the iron X-ray absorption K-edge. All data were indexed and processed using XDS and Aimless as part of the automatic xia2 pipeline.^42^ Reprocessing was carried out as necessary using Aimless as part of the CCP4 program suite.^43^ Statistics are summarized in Table S1 for X-ray data used for structure solution and refinement and in Table S2 for data used for calculation of Bijvoet-difference Fourier (anomalous-scattering density) maps.

Structure solution was performed by molecular replacement using phenix.phaser MR with the 1.7 Å resolution structure of wild type FtMt,^29^ pdb entry 1R03, as the search model. In all cases, the asymmetric unit contained a single copy of the protein monomer. Placement of iron ions followed reference to Bijvoet-difference Fourier maps calculated from anomalous scattering data collected at the peak of the iron K X-ray absorption edge (Table S2) and confirmed by the absence of corresponding peaks in similar maps calculated using anomalous scattering data collected on the low energy side of the same edge. After placing iron ions, bound magnesium ions were positioned at difference Fourier map peaks with reference to the indicators for metal ions described by Echols et al.^44^ Three iron sites in an iron-oxo cluster were found at peak heights above 4.0 σ in the anomalous difference electron density map calculated from data collected from FtMt/Fe^2+^ cocrystals exposed to aerobic well solution for 20 min before freezing. Two further iron sites (labeled 7 and 8) were located by MR-SAD^30^ and confirmed by ANODE.^31^ These were found at peak heights of 3.2 σ and 3.0 σ, respectively. At the resolution of the diffraction data available it was not possible to identify hydrogen atoms associated with the oxygen atoms in the vicinity of iron ions in the cluster, and thus the precise nature of the oxo species involved. Hence, oxygen atoms of water molecules were positioned with reference to difference Fourier maps and their placement checked by reference to omit maps which showed each oxygen to lie at a site at least 3σ above the map mean. However, we note that some peaks in the difference Fourier map are at short interatomic separations from their associated iron sites and likely represent oxide (O^2–^) coordinated to the metal. Model refinement employed iterative cycles using phenix.refine^45^ and manual correction using COOT.^46^ Anisotropic temperature factor refinement was employed for ferroxidase center ions and their occupancies were manually adjusted to ensure that the average *B* factor of the metal fell within ± 15% of the B factors of atoms of their environment. Statistics relating to the metal binding sites in the refined structures can be found in Table 1.

### Absorbance-Monitored Kinetic Studies

Protein activity was monitored via the increase in absorbance at 340 nm resulting from the oxidation of Fe^2+^ to Fe^3+^. The ability of variant proteins containing amino acid residue substitutions to form a mineral core was determined by monitoring the rate of increase in absorbance using a Hitachi U2900 spectrophotometer with sample chamber maintained at 25 °C. Ferrous ammonium sulfate dissolved in 1 mm HCl was added to a final concentration of 200 μm to a 0.5 μm solution of FtMt in 100 mm MES pH 6.5 in a 1 cm path length cuvette. The extinction coefficient of the mineral core was deduced from the net absorbance change once iron oxidation was complete. Initial rates of reaction were calculated from the slope of the linear region of a plot of absorbance at 340 nm vs time. Rapid iron oxidation at the FoC was monitored using an Applied Photophysics Bio-Sequential DX.17MV spectrophotometer with a 1 cm path length observation cell to mix equal volumes of 1 μm apo protein in 100 mm MES pH 6.5 and solutions of 6, 12, 18, 24, 30, 36, 42, 48, 60, 72, or 96 μm ferrous ammonium sulfate in 1 mm HCl. The time dependences of absorbance increases at 340 nm were fitted to the sum of two exponential processes, encompassing rapid (r) and slower (s) components, using OriginPro 8 (OriginLab):
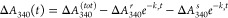
1

The extent to which oxidized iron vacates the FoCs was investigated by monitoring the regeneration of the rapid phase of iron oxidation associated with the apo protein. Fe^2+^ was added to a final concentration of 200 μm to 1 μm protein and absorbance at 340 nm monitored until it became invariant with time. This represented the end point of Fe^2+^ oxidation and, at this point, FoC binding sites were assumed saturated by Fe^3+^. Samples were then mixed by stopped flow with an equal volume of 72 μm Fe^2+^ in 1 mm HCl either immediately or following a further period of incubation of 3, 8, 15, or 60 min. An equivalent sample was incubated at 25 °C for 60 min followed by a further 15 h at 4 °C. Following re-equilibration at 25 °C the protein was mixed with an equal volume of 72 μm Fe^2+^ in 1 mm HCl as above. Traces were fitted to biexponential decay functions similar to that above but with a constant offset added representing the absorbance of 200 equiv of Fe^3+^ following the first addition, see Table S4. Comparison of the amplitude of the absorbance change associated with the rapid phase of Fe^3+^ oxidation to that of the corresponding apo protein mixed with 72 μm Fe^2+^ was used to estimate the percentage of vacant FoC sites at each delay time following the initial mineralization reaction (Table S4).

## Supporting Information Available

X-ray data collection and refinement statistics, datasets used for calculation of Bijvoet-difference Fourier maps, Occupancies of residue H65 in the refined structures, kinetic parameters for regeneration of rapid FoC activity, sequence comparison between mitochondrial and cytosolic H-chain ferritins, Structure of the ferroxidase center in anaerobically harvested FtMt/Fe^2+^ co-crystals, structure of the 3-fold channel in anaerobically harvested FtMt/Fe^2+^ co-crystals and corresponding structures following exposure to O_2_, structure of the iron binding site in the 4-fold channel of FtMt, structure of the ferroxidase center in FtMt/Fe^2+^ co-crystals exposed to aerobic well solution for 20 min prior to harvest, structures of H57A/E61A/E64A FtMt triple variant and HuHF focusing on the region corresponding to the nucleation site in wild-type FtMt, iron mineralization kinetics of H57A FtMt following pre-incubation with Fe^2+^, stopped-flow measurements of rapid iron oxidation catalysed by FtMt and inner surface variants, alignment of sequences of frog H’-chain, human H-chain and human mitochondrial ferritin, kinetic measurements demonstrating the effect of disrupting the proposed Fe^2+^ uptake route on rapid oxidation activity in FtMt (PDF)
xydata (XLSX)

## Notes

Final coordinates and structure factors were deposited in the Protein Data Bank (https://www.rcsb.org) with accession codes 9EQ9 (FtMt 0 min O_2_), 9EQ8 (FtMt 2 min O_2_), 9EQA (FtMt 20 min O_2_), 9EQB (H57A/E61A/E64A FtMt 20 min O_2_), 9EQC (HuHF 20 min O_2_).

## Abbreviations

FtMt: Mitochondrial ferritin
HuHF: Human H-chain ferritin
HuLF: Human L-chain ferritin
Bfr: Bacterioferritin

## References

[ref1] Bradley J. M.; Le Brun N. E.; Moore G. R. Ferritins: furnishing proteins with iron. J. Biol. Inorg. Chem. 2016, 21 (1), 13–28. 10.1007/s00775-016-1336-0. 26825805 PMC4771812

[ref2] Theil E. C. Ferritin protein nanocages use ion channels, catalytic sites, and nucleation channels to manage iron/oxygen chemistry. Curr. Opin Chem. Biol. 2011, 15 (2), 304–311. 10.1016/j.cbpa.2011.01.004. 21296609 PMC3074017

[ref3] Theil E. C.; Behera R. K.; Tosha T. Ferritins for chemistry and for life. Coord Chem. Revs 2013, 257 (2), 579–586. 10.1016/j.ccr.2012.05.013. 23470857 PMC3587046

[ref4] Crichton R. R.; Declercq J. P. X-ray structures of ferritins and related proteins. Biochim. Biophys. Acta 2010, 1800 (8), 706–718. 10.1016/j.bbagen.2010.03.019. 20363295

[ref5] Briat J. F.; Duc C.; Ravet K.; Gaymard F. Ferritins and iron storage in plants. Biochim. Biophys. Acta 2010, 1800 (8), 806–814. 10.1016/j.bbagen.2009.12.003. 20026187

[ref6] Le Brun N. E.; Crow A.; Murphy M. E. P.; Mauk A. G.; Moore G. R. Iron core mineralisation in prokaryotic ferritins. Biochim. Biophys. Acta 2010, 1800 (8), 732–744. 10.1016/j.bbagen.2010.04.002. 20388533

[ref7] Almiron M.; Link A. J.; Furlong D.; Kolter R. A novel DNA-binding protein with regulatory and protective roles in starved *Escherichia coli*. Genes Dev. 1992, 6 (12B), 2646–2654. 10.1101/gad.6.12b.2646. 1340475

[ref8] Bradley J. M.; Moore G. R.; Le Brun N. E. Diversity of Fe^2+^ entry and oxidation in ferritins. Curr. Opin Chem. Biol. 2017, 37, 122–128. 10.1016/j.cbpa.2017.02.027. 28314217

[ref9] Masuda T.; Goto F.; Yoshihara T.; Mikami B. Crystal structure of plant ferritin reveals a novel metal binding site that functions as a transit site for metal transfer in ferritin. J. Biol. Chem. 2010, 285 (6), 4049–4059. 10.1074/jbc.M109.059790. 20007325 PMC2823546

[ref10] Johnson E.; Cascio D.; Sawaya M. R.; Gingery M.; Schroder I. Crystal structures of a tetrahedral open pore ferritin from the hyperthermophilic Archaeon *Archaeoglobus fulgidus*. Structure 2005, 13 (4), 637–648. 10.1016/j.str.2005.01.019. 15837202

[ref11] Bou-Abdallah F. The iron redox and hydrolysis chemistry of the ferritins. Biochim. Biophys. Acta 2010, 1800 (8), 719–731. 10.1016/j.bbagen.2010.03.021. 20382203

[ref12] Wade V. J.; Levi S.; Arosio P.; Treffry A.; Harrison P. M.; Mann S. Influence of site-directed modifications on the formation of iron cores in ferritin. J. Mol. Biol. 1991, 221 (4), 1443–1452. 10.1016/0022-2836(91)90944-2. 1942061

[ref13] Theil E. C.; Tosha T.; Beherat R. K. Solving biology’s iron chemistry problem with ferritin protein nanocages. Acc. Chem. Res. 2016, 49 (5), 784–791. 10.1021/ar500469e. 27136423

[ref14] Bradley J. M.; Svistunenko D. A.; Wilson M. T.; Hemmings A. M.; Moore G. R.; Le Brun N. E. Bacterial iron detoxification at the molecular level. J. Biol. Chem. 2020, 295 (51), 17602–17623. 10.1074/jbc.REV120.007746. 33454001 PMC7762939

[ref15] Levi S.; Corsi B.; Bosisio M.; Invernizzi R.; Volz A.; Sanford D.; Arosio P.; Drysdale J. A human mitochondrial ferritin encoded by an intronless gene. J. Biol. Chem. 2001, 276 (27), 24437–24440. 10.1074/jbc.C100141200. 11323407

[ref16] Campanella A.; Rovelli E.; Santambrogio P.; Cozzi A.; Taroni F.; Levi S. Mitochondrial ferritin limits oxidative damage regulating mitochondrial iron availability: hypothesis for a protective role in Friedreich ataxia. Hum. Mol. Genet. 2008, 18 (1), 1–11. 10.1093/hmg/ddn308. 18815198 PMC3298861

[ref17] Snyder A. M.; Neely E. B.; Levi S.; Arosio P.; Connor J. R. Regional and cellular distribution of mitochondrial ferritin in the mouse brain. J. Neurosci Res. 2010, 88 (14), 3133–3143. 10.1002/jnr.22462. 20722075

[ref18] Mendsaikhan A.; Takeuchi S.; Walker D. G.; Tooyama I. Differences in gene expression profiles and phenotypes of differentiated SH-SY5Y neurons stably overexpressing mitochondrial ferritin. Front Mol. Neurosci 2019, 11, 470 10.3389/fnmol.2018.00470. 30670947 PMC6331485

[ref19] Bradley J. M.; Bugg Z.; Pullin J.; Moore G. R.; Svistunenko D. A.; Le Brun N. E. Human mitochondrial ferritin exhibits highly unusual iron-O_2_ chemistry distinct from that of cytosolic ferritins. BioRxiv. 2025-03-31. DOI: 10.1101/2025.03.31.646379. PMC12092714 40393986

[ref20] Ciambellotti S.; Pozzi C.; Mangani S.; Turano P. Iron biomineral growth from the initial nucleation seed in L-ferritin. Chem. - Eur. J. 2020, 26 (26), 5770–5773. 10.1002/chem.202000064. 32027764

[ref21] Pozzi C.; Ciambellotti S.; Bernacchioni C.; Di Pisa F.; Mangani S.; Turano P. Chemistry at the protein-mineral interface in L-ferritin assists the assembly of a functional (μ_3_-oxo)Tris (μ_2_-peroxo) triiron(III) cluster. Proc. Natl. Acad. Sci. U. S. A. 2017, 114 (10), 2580–2585. 10.1073/pnas.1614302114. 28202724 PMC5347543

[ref22] Galvez N.; Fernandez B.; Sanchez P.; Cuesta R.; Ceolin M.; Clemente-Leon M.; Trasobares S.; Lopez-Haro M.; Calvino J. J.; Stephan O.; Dominguez-Vera J. M. Comparative structural and chemical studies of ferritin cores with gradual removal of their iron contents. J. Am. Chem. Soc. 2008, 130 (25), 8062–8068. 10.1021/ja800492z. 18507465

[ref23] Jobichen C.; Ying Chong T.; Rattinam R.; Basak S.; Srinivasan M.; Choong Y. K.; Pandey K. P.; Ngoc T. B.; Shi J.; Angayarkanni J.; Sivaraman J. Bacterioferritin nanocage structures uncover the biomineralization process in ferritins. PNAS Nexus 2023, 2 (7), pgad235 10.1093/pnasnexus/pgad235. 37529551 PMC10388152

[ref24] Gayer K. H.; Woontner L. The solubility of ferrous hydroxide and ferric hydroxide in acidic and basic media at 25-degrees. J. Phys. Chem. 1956, 60 (11), 1569–1571. 10.1021/j150545a021.

[ref25] Pozzi C.; Di Pisa F.; Lalli D.; Rosa C.; Theil E.; Turano P.; Mangani S. Time-lapse anomalous X-ray diffraction shows how Fe^2+^ substrate ions move through ferritin protein nanocages to oxidoreductase sites. Acta Crystallogr. D Struct Biol. 2015, 71, 941–953. 10.1107/S1399004715002333. PMC4388269 25849404

[ref26] Bertini I.; Lalli D.; Mangani S.; Pozzi C.; Rosa C.; Theil E. C.; Turano P. Structural insights into the ferroxidase site of ferritins from higher eukaryotes. J. Am. Chem. Soc. 2012, 134 (14), 6169–6176. 10.1021/ja210084n. 22424302 PMC4159105

[ref27] Pozzi C.; Di Pisa F.; Bernacchioni C.; Ciambellotti S.; Turano P.; Mangani S. Iron binding to human heavy-chain ferritin. Acta Crystallogr. D Struct Biol. 2015, 71, 1909–1920. 10.1107/S1399004715013073. 26327381

[ref28] Ciambellotti S.; Pratesi A.; Tassone G.; Turano P.; Mangani S.; Pozzi C. Iron binding in the ferroxidase site of human mitochondrial ferritin. Chem. - Eur. J. 2021, 27 (59), 14690–14701. 10.1002/chem.202102270. 34343376

[ref29] Langlois d'Estaintot B.; Santambrogio P.; Granier T.; Gallois B.; Chevalier J. M.; Précigoux G.; Levi S.; Arosio P. Crystal structure and biochemical properties of the human mitochondrial ferritin and its mutant Ser144Ala. J. Mol. Biol. 2004, 340 (2), 277–293. 10.1016/j.jmb.2004.04.036. 15201052

[ref30] Bou-Abdallah F.; Santambrogio P.; Levi S.; Arosio P.; Chasteen N. D. Unique iron binding and oxidation properties of human mitochondrial ferritin: A comparative analysis with human H-chain ferritin. J. Mol. Biol. 2005, 347 (3), 543–554. 10.1016/j.jmb.2005.01.007. 15755449

[ref31] Read R. J.; McCoy A. J. Maximum-likelihood determination of anomalous substructures. Acta Crystallogr., Sect. D: Biol. Crystallogr. 2018, 74 (Pt 2), 98–105. 10.1107/S2059798317013468. PMC5947773 29533235

[ref32] Thorn A.; Sheldrick G. M. ANODE: anomalous and heavy-atom density calculation. J. Appl. Crystallogr. 2011, 44 (Pt 6), 1285–1287. 10.1107/S0021889811041768. 22477786 PMC3246834

[ref33] Kumar K. S. D.; Gurusaran M.; Satheesh S. N.; Radha P.; Pavithra S.; Thulaa Tharshan K. P. S.; Helliwell J. R.; Sekar K. Online_DPI: a web server to calculate the diffraction precision index for a protein structure. J. Appl. Crystallogr. 2015, 48, 939–942. 10.1107/S1600576715006287.

[ref34] Michel F. M.; Ehm L.; Antao S. M.; Lee P. L.; Chupas P. J.; Liu G.; Strongin D. R.; Schoonen M. A.; Phillips B. L.; Parise J. B. The structure of ferrihydrite, a nanocrystalline material. Science 2007, 316 (5832), 1726–1729. 10.1126/science.1142525. 17525301

[ref35] Levi S.; Salfeld J.; Franceschinelli F.; Cozzi A.; Dorner M. H.; Arosio P. Expression and structural and functional properties of human ferritin L-chain from Escherichia coli. Biochemistry 1989, 28 (12), 5179–5184. 10.1021/bi00438a040. 2669970

[ref36] Bradley J. M.; Pullin J.; Moore G. R.; Svistunenko D. A.; Hemmings A. M.; Le Brun N. E. Routes of iron entry into, and exit from, the catalytic ferroxidase sites of the prokaryotic ferritin SynFtn. Dalton Trans 2020, 49 (5), 1545–1554. 10.1039/C9DT03570B. 31930254

[ref37] Behera R. K.; Theil E. C. Moving Fe^2+^ from ferritin ion channels to catalytic OH centers depends on conserved protein cage carboxylates. Proc. Natl. Acad. Sci. U. S. A. 2014, 111 (22), 7925–7930. 10.1073/pnas.1318417111. 24843174 PMC4050572

[ref38] Tosha T.; Ng H. L.; Bhattasali O.; Alber T.; Theil E. C. Moving metal ions through ferritin-protein nanocages from three-fold pores to catalytic sites. J. Am. Chem. Soc. 2010, 132 (41), 14562–14569. 10.1021/ja105583d. 20866049 PMC3211085

[ref39] Behera R. K.; Torres R.; Tosha T.; Bradley J. M.; Goulding C. W.; Theil E. C. Fe^2+^ substrate transport through ferritin protein cage ion channels influences enzyme activity and biomineralization. J. Biol. Inorg. Chem. 2015, 20 (6), 957–969. 10.1007/s00775-015-1279-x. 26202907 PMC4634868

[ref40] Bradley J. M.; Fair J.; Hemmings A. M.; Le Brun N. E. Key carboxylate residues for iron transit through the prokaryotic ferritin SynFtn. Microbiology 2021, 167 (11), 001105 10.1099/mic.0.001105. 34825885 PMC8743623

[ref41] Bauminger E. R.; Harrison P. M.; Hechel D.; Nowik I.; Treffry A. Mssbauer spectroscopic investigation of sructure-function relations in ferritins. Biochim. Biophys. Acta 1991, 1118 (1), 48–58. 10.1016/0167-4838(91)90440-B. 1764477

[ref42] Winter G. xia2: an expert system for macromolecular crystallography data reduction. J. Appl. Crystallogr. 2010, 43, 186–190. 10.1107/S0021889809045701.

[ref43] Winn M. D.; Ballard C. C.; Cowtan K. D.; Dodson E. J.; Emsley P.; Evans P. R.; Keegan R. M.; Krissinel E. B.; Leslie A. G. W.; McCoy A.; et al. Overview of the CCP4 suite and current developments. Acta Crystallogr. D Struct Biol. 2011, 67, 235–242. 10.1107/S0907444910045749. PMC3069738 21460441

[ref44] Echols N.; Morshed N.; Afonine P. V.; McCoy A. J.; Miller M. D.; Read R. J.; Richardson J. S.; Terwilliger T. C.; Adams P. D. Automated identification of elemental ions in macromolecular crystal structures. Acta Crystallogr., Sect. D: Biol. Crystallogr. 2014, 70 (Pt 4), 1104–1114. 10.1107/S1399004714001308. 24699654 PMC3975891

[ref45] Afonine P. V.; Grosse-Kunstleve R. W.; Echols N.; Headd J. J.; Moriarty N. W.; Mustyakimov M.; Terwilliger T. C.; Urzhumtsev A.; Zwart P. H.; Adams P. D. Towards automated crystallographic structure refinement with phenix.refine. Acta Crystallogr. D Struct Biol. 2012, 68, 352–367. 10.1107/S0907444912001308. PMC3322595 22505256

[ref46] Emsley P.; Lohkamp B.; Scott W. G.; Cowtan K. Features and development of Coot. Acta Crystallogr. D Biol. Crystallogr. 2010, 66, 486–501. 10.1107/S0907444910007493. 20383002 PMC2852313

