# Supplementary material for: Observation of the Assembly of the Nascent Mineral Core at the Nucleation Site of Human Mitochondrial Ferritin
Source: J Am Chem Soc. 2025 Apr 14;147(16):13699–710. doi: 10.1021/jacs.5c01337 (PMC12022971; doi:10.1021/jacs.5c01337)
Supplement: Supplementary file 1 — ja5c01337_si_001.pdf [file ja5c01337_si_001.pdf]

Supporting Information

Observation of the assembly of the nascent mineral core at the nucleation site of human mitochondrial ferritin

Justin M. Bradley, Zinnia Bugg, Geoffrey R. Moore, Andrew M. Hemmings and Nick E. Le Brun

Supporting Tables

Table S1. X-ray data collection and refinement statistics[&].

**Sample**	**0min O_2 soak**	**2min O_2 soak**	**20min O_2 soak**	**AAA***	**HuHF[$]**
PDB entry code	9EQ9	9EQ8	9EQA	9EQB	9EQC
Beamline	DLS I03	DLS I03	DLS I03	DLS I04	DLS I04
Wavelength/ Å	0.9763	0.9763	1.7395	0.9763	0.9763
Resolution range	51.22 - 1.84 (1.91 - 1.84)	40.87 - 1.48 (1.53 - 1.48)	91.26 - 1.97 (2.04 - 1.97)	52.65 - 1.36 (1.41 - 1.36)	45.69 - 1.60 (1.66 - 1.60)
Space group	F 4 3 2	F 4 3 2	F 4 3 2	F 4 3 2	F 4 3 2
Unit cell	177.43	182.77	182.53	182.40	182.74
Total reflections	454449 (46407)	739734 (22155)	602555 (24073)	1340404 (68449)	922702 (93223)
Unique reflections	21331 (2088)	43961 (4286)	19012 (1862)	56043 (5464)	35006 (3432)
Multiplicity	21.3 (22.2)	16.8 (5.2)	31.7 (12.9)	23.9 (12.5)	26.4 (27.1)
Completeness (%)	99.95 (99.90)	99.91 (99.44)	95.89 (58.98)	99.84 (98.48)	99.83 (98.89)
Mean I/sigma(I)	16.76 (0.85)	17.27 (1.00)	11.82 (0.30)	9.56 (0.45)	11.88 (0.45)
Wilson B-factor	34.81	19.33	40.42	15.91	27.76
R-merge	0.150 (4.838)	0.147 (2.473)	0.151 (2.424)	0.207 (2.278)	0.165 (1.802)
R-meas	0.154 (4.952)	0.151 (2.723)	0.154 (2.524)	0.212 (2.376)	0.168 (1.836)
R-pim	0.033 (1.045)	0.034 (1.093)	0.026 (0.681)	0.042 (0.661)	0.032 (0.351)
CC1/2	0.999 (0.364)	0.999 (0.391)	0.999 (0.462)	0.998 (0.355)	0.999 (0.640)
CC*	1 (0.730)	1 (0.750)	1 (0.795)	1 (0.724)	1 (0.884)
Reflections used in refinement	21327 (2086)	43955 (4281)	18242 (1103)	55955 (5381)	34953 (3400)
Reflections used for R-free	1080 (117)	2107 (200)	929 (66)	2807 (265)	1755 (164)
R-work	0.199 (0.311)	0.175 (0.319)	0.207 (0.370)	0.175 (0.342)	0.191 (0.518)
R-free	0.241 (0.390)	0.189 (0.317)	0.244 (0.385)	0.201 (0.365)	0.238 (0.532)
CC(work)	0.915 (0.685)	0.951 (0.651)	0.933 (0.649)	0.959 (0.725)	0.955 (0.848)
CC(free)	0.916 (0.411)	0.957 (0.630)	0.917 (0.659)	0.949 (0.639)	0.869 (0.722)
Number of non-hydrogen atoms	1456	1571	1454	1627	1608
macromolecules	1355	1372	1362	1369	1426
ligands	22	21	14	13	14
solvent	79	178	78	245	168
Protein residues	166	168	166	167	172
RMS(bonds)	0.012	0.009	0.012	0.005	0.009
RMS(angles)	1.24	1.09	1.24	0.81	1.03
Ramachandran favored (%)	97.56	98.8	98.17	98.79	98.24
Ramachandran allowed (%)	2.44	1.2	1.83	1.21	1.76
Ramachandran outliers (%)	0	0	0	0	0
Rotamer outliers (%)	0	0	0.68	0.69	0.65
Clashscore	7.87	3.71	2.61	3.71	3.92
Average B-factor ($Å^2$)	43.08	27.54	50.37	21.6	28.23
macromolecules	42.39	25.75	49.96	18.55	26.81
ligands	55.97	46.61	60.81	35.46	47.85
solvent	51.33	39.12	55.63	37.88	38.61

[&]Data in brackets refers to highest resolution shell. *H57A/E61A/E64A FtMt triple variant 20 min O_2 soak. [$]Human H-chain ferritin 20 min O_2 soak.

Table S2. Statistics for datasets used for calculation of Bijvoet-difference Fourier maps.

	**0 min O_2 soak**	**2 min O_2 soak**	**20 min O_2 soak**	**AAA***	**HuHF$**
Beamline	DLS I03	DLS I03	DLS I03	DLS I04	DLS I04
Wavelength/ Å	1.7395	1.7395	1.7395	1.7396	1.7396
Resolution range	63.0 - 2.52	64.7 - 2.38	91.3 - 1.97	105.3 - 1.94	91.4 - 2.10
Total reflections	632330	807453	602555	1023231	1000553
Unique reflections	8697	31680	19012	18863	15418
Multiplicity	72.7	25.5	31.7	54.3	64.9
Completeness (%)	100.0	100.0	95.9	95.1	97.3
Anomalous multi-plicity	40.7	13.40	37.90	29.6	31.5
Anomalous com-pleteness (%)	99.9	100.0	100.0	92.3	86.9
<I>	93.1	60.4	57.6	59.4	67.8
<I/σI>	12.3	10.4	11.8	44.2	43.1
Rmerge	0.521	0.333	0.151	0.090	0.098
Rmeas	0.525	0.340	0.154	0.090	0.099
Rpim	0.061	0.068	0.026	0.011	0.012
CC(1/2)	0.999	0.998	0.999	0.999	1.000
CC(anom)	0.524	0.328	0.539	0.796	0.794
&Anomalous signal resolution limit/ Å	3.64	3.61	2.51	2.03	2.12

*H57A/E61A/E64A FtMt triple variant 20min O_2 soak. $Human H-chain ferritin 20min O_2 soak. &Resolution limit of the anomalous signal calculated by Aimless [1].

Table S3. Occupancies of residue H65 in the refined structures.

	**Occupancy**
**0 min**	0.72, 0.28
**2 min**	0.72, 0.28
**20 min**	0.47, 0.53
**AAA***	0.57, 0.43
**HuHF$**	0.62, 0.38

*H57A/E61A/E64A FtMt triple variant 20 min O_2 soak. $Human H-chain ferritin 20 min O_2 soak.

Table S4. Kinetic parameters for regeneration of rapid FoC activity*.

	**Protein**				
	wild type	H57A	E61A	E64A	H57A/E61A/E64A
$A(r)_{apo}$	0.045	0.040	0.047	0.047	0.050
$k(r)_{apo}$	10.4 s^{-1}	15.2 s^{-1}	4.6 s^{-1}	4.8 s^{-1}	3.3 s^{-1}
$A(r)_{0\ min}$	0.0048	0.0024	-	0.0047	-
$k(r)_{0\ min}$	3.8 s^{-1}	3.8 s^{-1}	-	1.9 s^{-1}	-
$A(r)_{3\ min}$	0.010	0.0068	-	0.0092	-
$k(r)_{3\ min}$	3.4 s^{-1}	2.7 s^{-1}	-	2.0 s^{-1}	-
$A(r)_{8\ min}$	0.014	0.011	-	0.013	-
$k(r)_{8\ min}$	3.7 s^{-1}	4.0 s^{-1}	-	1.9 s^{-1}	-
$A(r)_{15\ min}$	0.017	0.015	0.0035	0.015	-
$k(r)_{15\ min}$	4.2 s^{-1}	5.0 s^{-1}	2.2 s^{-1}	2.2 s^{-1}	-
$A(r)_{60\ min}$	0.020	0.018	0.0093	0.018	0.0080
$k(r)_{60\ min}$	5.2 s^{-1}	6.0 s^{-1}	1.8 s^{-1}	3.0 s^{-1}	1.3 s^{-1}
$A(r)_{overnight}$	0.021	0.020	0.019	0.018	0.021
$k(r)_{overnight}$	10.0 s^{-1}	11.2 s^{-1}	3.0 s^{-1}	5.8 s^{-1}	2.7 s^{-1}

*A(r) is the amplitude associated with the rapid phase of Fe^{2+} oxidation and k(r) the apparent first order rate constant for this phase. Parameters are reported for the oxidation observed following addition of 72 Fe^{2+} ions to apo proteins, and to proteins incubated for the indicated times following the oxidation of an initial aliquot of 200 equivalents of Fe^{2+}. The time dependences of absorbance at 340 nm were fitted to the sum of two exponential processes, encompassing rapid (r) and slower (s) components, using OriginPro 8 (OriginLab) and Equation 1 for apo proteins, and Equation S1 for proteins already containing iron:

$$A_{340}(t) = A_i + \Delta A_{340}^{(tot)} - \Delta A_{340}^{r} e^{-k_r t} - \Delta A_{340}^{s} e^{-k_s t} \qquad \textit{Equ. S1}$$

In Equation S1, A_i is the starting absorbance following oxidation of the initial addition of 200 equivalents of Fe^{2+}. The extent (percentage) to which regeneration of rapid oxidation activity had occurred after delay time t was determined using Equation S2:

$$\%regeneration(t) = 100 \times A_{rapid}(t)/A_{rapid}(apo) \qquad \textit{Equ. S2}$$

Where $A_{rapid}(apo)$ is the amplitude associated with rapid oxidation of Fe^{2+} in apo FtMt.

Supporting Figures

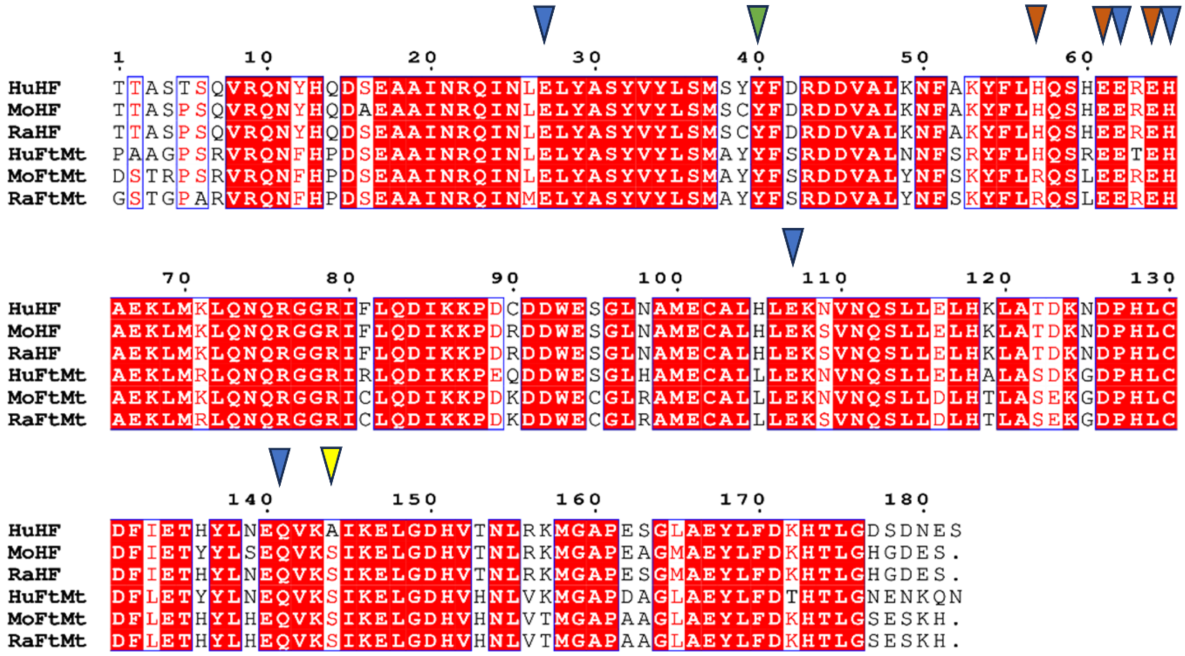

Figure S1. Comparison between mitochondrial and cytosolic H-chain ferritins. Alignment of amino acid residue sequences of mitochondrial ferritins with human H-chain ferritins. Conserved residues are indicated in red. The N-terminal mitochondrial targeting sequences of the mitochondrial ferritins are omitted from the alignments. Blue arrowheads indicate conserved ferroxidase center residues. The green arrowhead identifies the conserved Tyr residue lying close to the ferroxidase center that plays a key mechanistic role in rapid Fe^{2+} oxidation. Brown arrowheads indicate the inner surface residues that interact with the iron-oxo cluster identified in this work. The yellow arrowhead indicates the position of Ser144 in mitochondrial and some cytosolic H-chain ferritins, but which is substituted by Ala144 in human H-chain ferritin. The red double-headed arrow indicates the position of the amino acid residues lining the channel. The nine N-terminal amino acids of the human FtMt sequence depicted above were absent from the wild-type and variant proteins reported in this study. The alignment was performed using Clustal Omega [2] and presented using ESPript 3.0 [3].

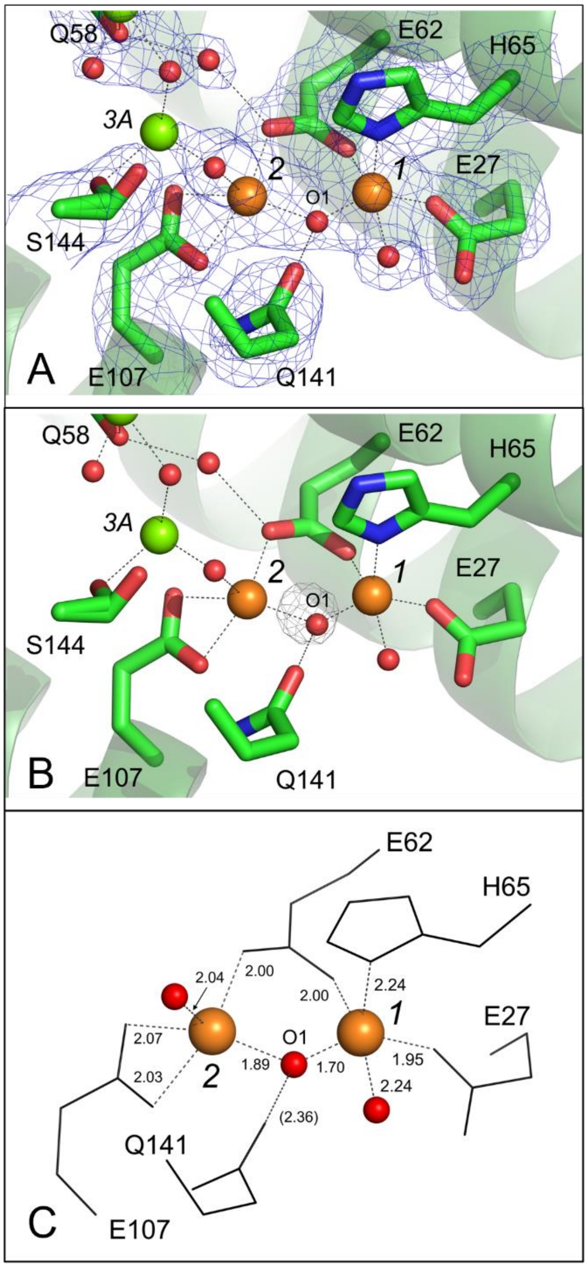

Figure S2. The ferroxidase center in anaerobically harvested FtMt/Fe^{2+} co-crystals. (**A**) Blue mesh shows the Sigma-A weighted Fourier (2mFo-DFc) map contoured at 1.1 σ. Iron positions are indicated by orange spheres, oxygen by red spheres. Individual residues are shown in stick format and labelled. Metal binding sites are indicated by italicised black numbers and metal coordination bonds are shown as black dashed lines. The single oxygen site bridging the ferroxidase iron positions is labelled O1. (**B**) as (A) but grey mesh shows the O1 oxygen atom omit map contoured at 6σ. (**C**) as (A) but showing detail of the geometry of the ferroxidase center. Individual residues are shown in wire format and labelled. The lengths of metal coordination bonds are shown in Ångstrom units. The length of the hydrogen bond from the sidechain of Q141 to O1 is indicated in brackets. Other geometry values are provided in Table 2.

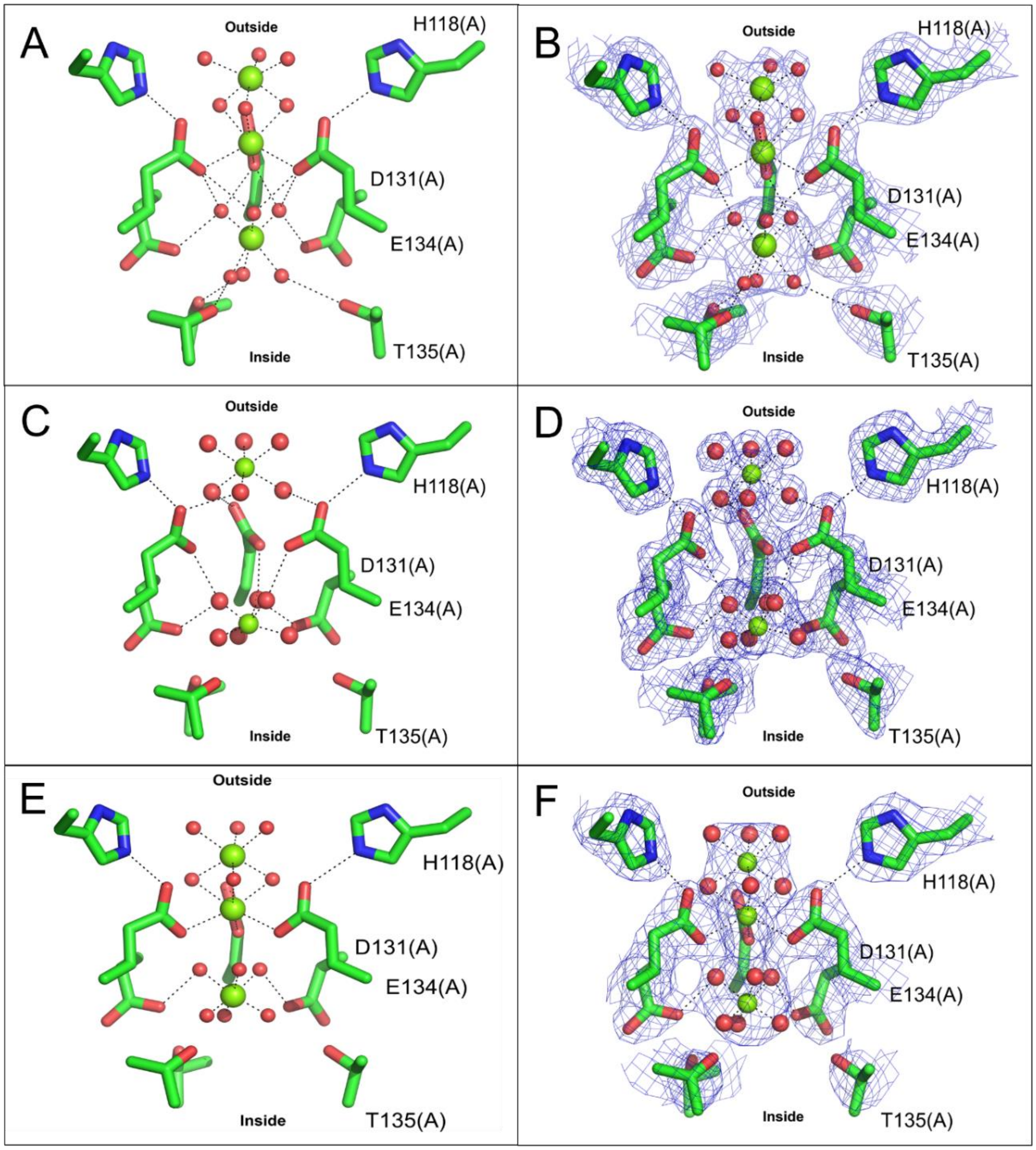

Figure S3. (**A**) **View of the 3-fold channel in anaerobically harvested FtMt/Fe^{2+} co-crystals**. Magnesium ion positions are indicated by green spheres, oxygen by red spheres. Individual residues lining the channel are shown in stick format and those of one protein subunit are labelled. Note that single instances of residues H118 and D131 have been removed for clarity. Metal coordination and hydrogen bonds are shown as black dashed lines. The inside and outside of the FtMt cavity are indicated. (**B**) as (A) but the blue mesh shows the Sigma-A weighted Fourier (2mFo-DFc) map contoured at 1.1σ. (**C**, **D**) as (A, B) but for co-crystals exposed to aerobic cryoprotectant for 2 min prior to harvesting. (**E**, **F**) as (A, B) but for co-crystals exposed to aerobic cryoprotectant for 20 min prior to harvesting.

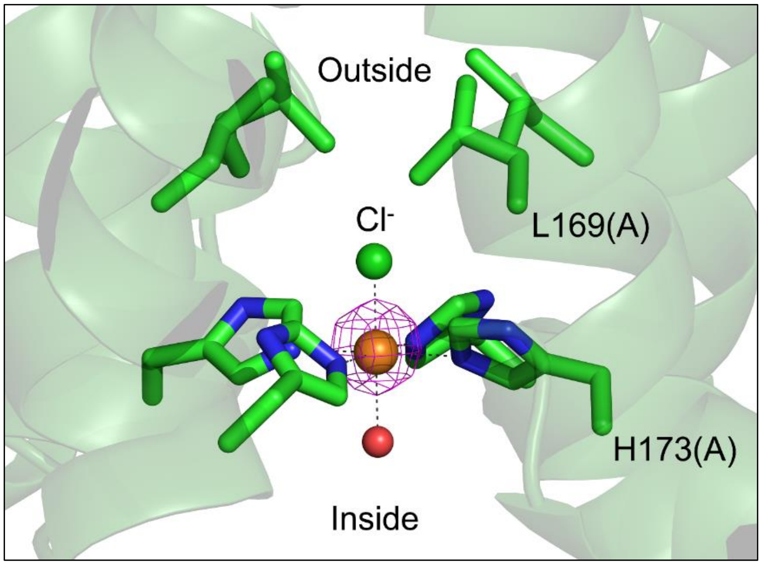

Figure S4. An iron binding site in the 4-fold channel of FtMt. Octahedrally-coordinated iron (orange sphere) located at a site in the 4-fold channel found in anaerobically harvested FtMt/Fe^{2+} co-crystals. Magenta mesh shows the anomalous difference Fourier map contoured at 10 σ in the vicinity of the iron ion. This site lies on the crystallographic 4-fold axis and persists following exposure of crystals to O_2. The iron ion is coordinated axially by four imidazole ε-nitrogen atoms of symmetry-related copies of residue H173. These residues are shown in stick format and labelled as are L169, another residue lining the channel. Metal coordination bonds are shown as black dashed lines. A chloride anion and a water molecule occupy the axial coordination positions of the metal ion and are directed towards the external and the internal surface of the cage, respectively.

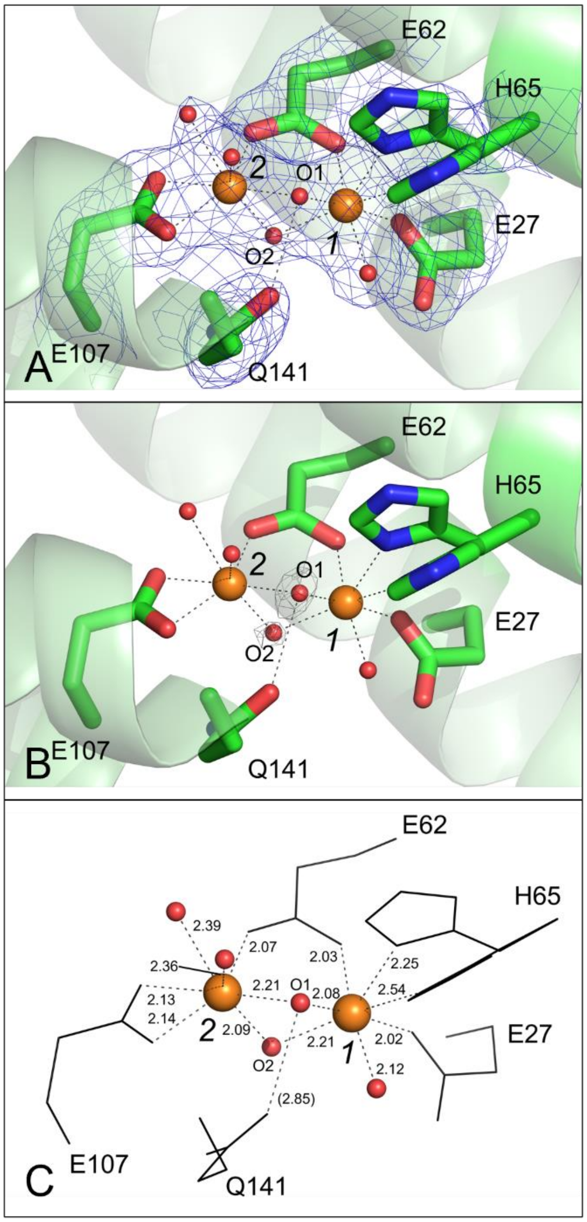

Figure S5. The ferroxidase center in FtMt/Fe^{2+} co-crystals exposed to aerobic well solution for 20 min prior to harvest. (**A**) Blue mesh shows the Sigma-A weighted Fourier (2mFo-DFc) map contoured at 1.1 σ. Iron positions are indicated by orange spheres, oxygen by red spheres. Individual residues are shown in stick format and labelled. Metal-binding sites are indicated by italicised black numbers and metal coordination bonds are shown as black dashed lines. The oxygen atoms bridging the ferroxidase iron positions are labelled O1 and O2. (**B**) as (A) but grey mesh shows the oxygen omit map contoured at 6σ. (**C**) as (A) but showing detail of the geometry of the FoC. Individual residues are shown in wire format and labelled. The lengths of metal coordination bonds are shown in Ångstrom units. The length of the hydrogen bond from the sidechain of Q141 to atom O1 is indicated in brackets. Other geometry values are provided in Table 2.

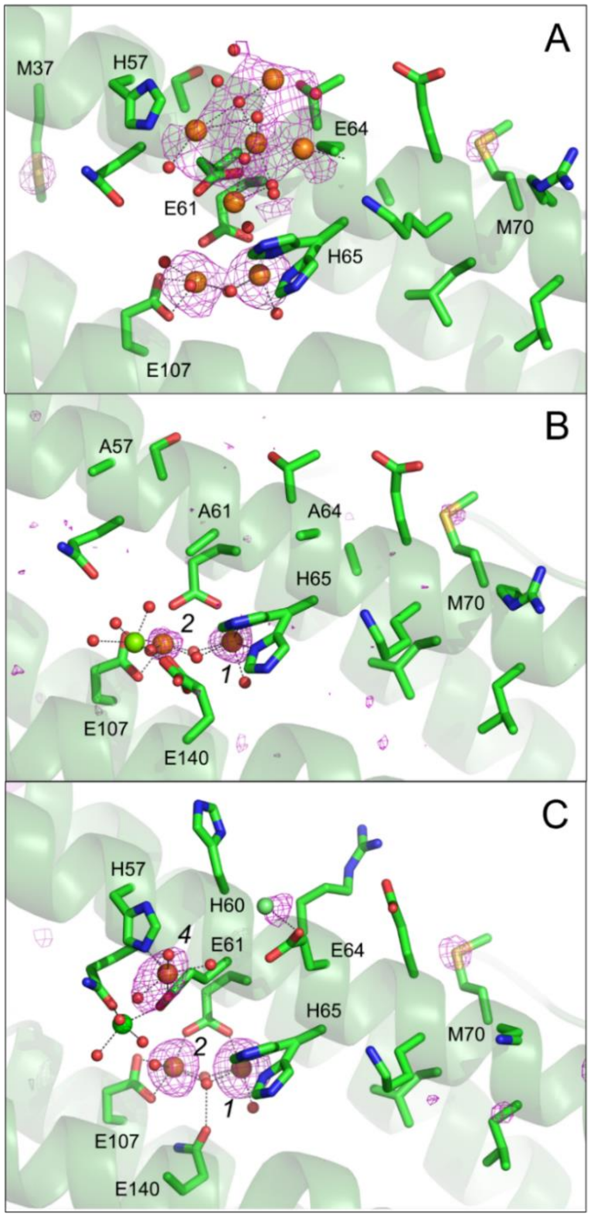

Figure S6. The H57A/E61A/E64A FtMt triple variant and HuHF do not form an iron-oxo cluster. (**A**) A view showing the environment of the iron-oxo cluster and ferroxidase center in FtMt/Fe^{2+} co-crystals exposed to aerobic well solution for 20 min before harvesting. The magenta mesh shows the anomalous difference Fourier map contoured at 3 σ. Iron positions are indicated by orange spheres, oxygen by red spheres. Metal coordination bonds are shown as black dashed lines. Selected residues are shown in stick format and labelled. Note that the side chain of residue Glu64 is presumed disordered as no significant electron density beyond atom Cγ was observed. (**B**) as (A) but for H57A/E61A/E64A FtMt triple variant/Fe^{2+} co-crystals exposed to aerobic cryoprotectant for 20 min before harvesting. A magnesium ion position is shown by a green sphere. While the expected peaks in the anomalous difference Fourier map for the iron positions of the ferroxidase center and for methionine sulfur atoms are present, no evidence for an iron-oxo cluster was observed. (**C**) as (A, B) but for HuHF/Fe^{2+} co-crystals exposed to aerobic cryoprotectant for 20 min before harvesting. In the case of human H-chain ferritin, an iron ion was found at site 4 and a chloride ion is found in the vicinity of E64 (shown as a light green sphere) but, as for the H57A/E61A/E64A FtMt triple variant, no evidence was found in the anomalous difference Fourier map for an iron-oxo cluster.

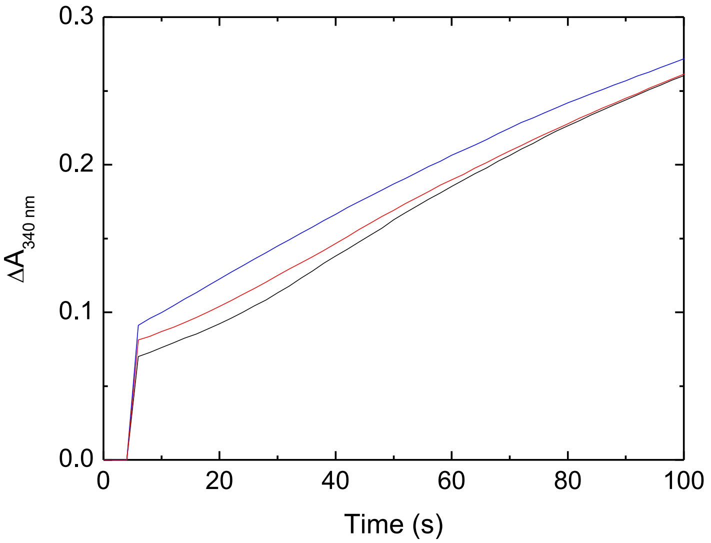

Figure S7. Pre-incubation of variant H57A with Fe^{2+} abolished the lag phase in iron mineralization. Increases in 340 nm absorbance for 0.5 μM solutions of variant H57A FtMt pre-incubated with 24 (black), 48 (red) or 72 (blue) equivalents of Fe^{2+} prior to the addition of a further 400 equivalents are shown. The data show that addition and oxidation of 72 equivalents (3 per monomer) prior to initiating the mineralization assay was sufficient to eliminate the initial lower rate of activity, and thus to rescue the behaviour to resemble that of the wild-type protein.

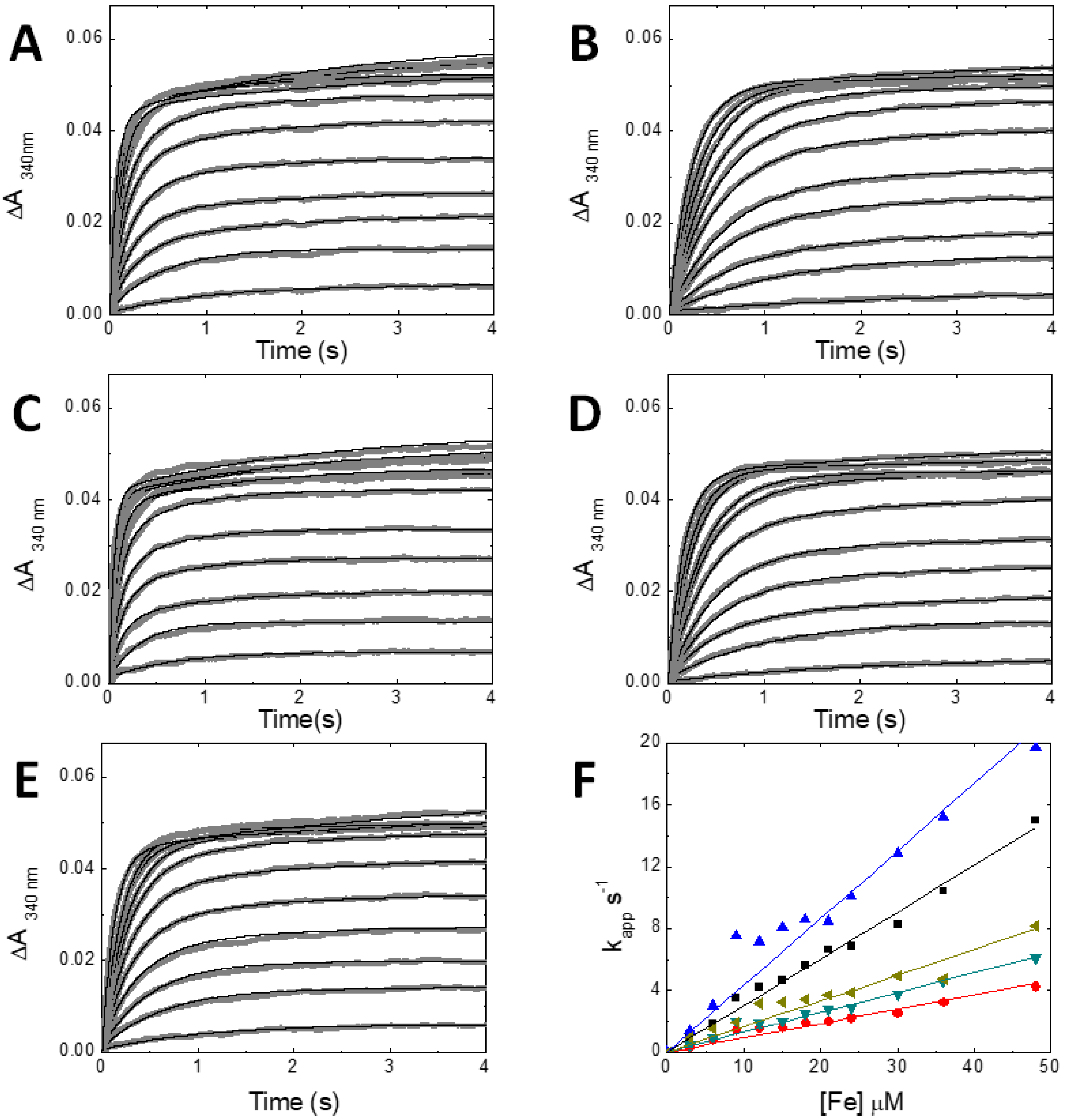

Figure S8. Rapid iron oxidation by FtMt and inner surface variants. The increase in absorbance at 340 nm following aerobic mixing of with Fe^{2+} with (**A**) wild-type, or variants H57A/E61A/E64A (**B**), H57A (**C**), E61A (**D**) and E64A (**E**) to give final protein concentrations of 0.5 μM and metal ion concentration between 3 and 48 μM. Data, represented by grey circles, are fitted to a bi-exponential decay (black traces) describing rapid and slow phases of iron oxidation. Panel (**F**) shows the linear dependence of the pseudo first-order rate constants associated with the rapid phase of Fe^{2+} oxidation on Fe^{2+} concentration for wild type (black), H57A/E61A/E64A (red), H57A (blue), E61A (dark cyan) and E64A (dark yellow) FtMt. The gradient of each plot is the apparent second-order rate constant (with units of M^{-1} s^{-1}), see Table 3 of the main paper.

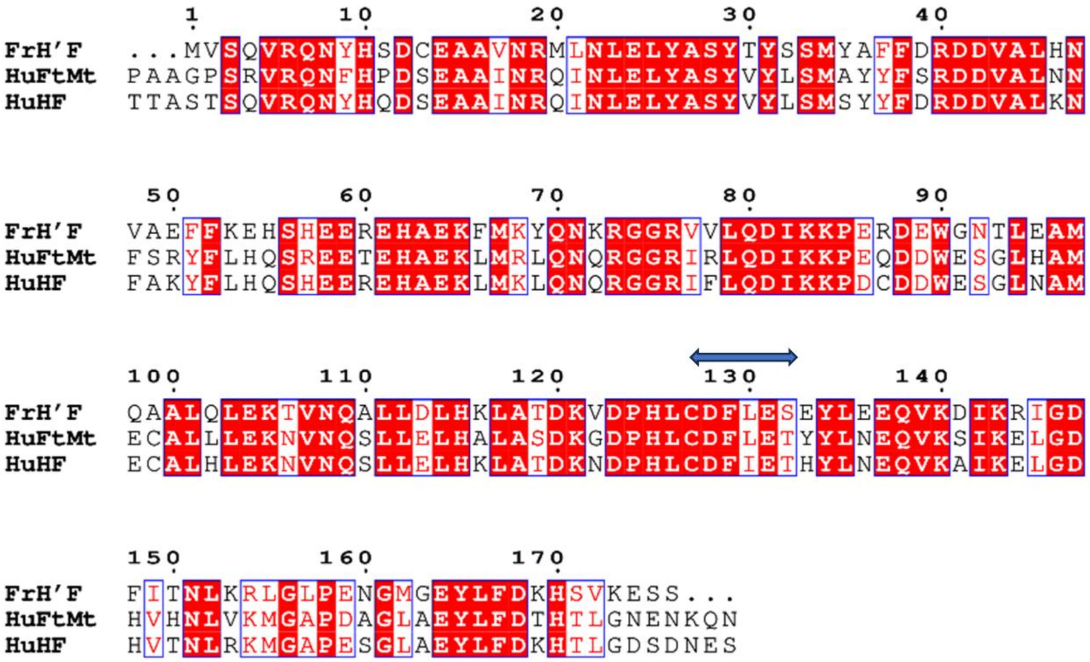

Figure S9. Alignment of sequences of frog H'-chain, human H-chain and human mitochondrial ferritin. The 3-fold channel of frog H' ferritin has been demonstrated to be the major route of iron uptake [4]. Alignment with FtMt and HuHF shows a high level of sequence identity/similarity in this region of the proteins. The blue double-headed arrow indicates the position of the amino acids lining the channel.

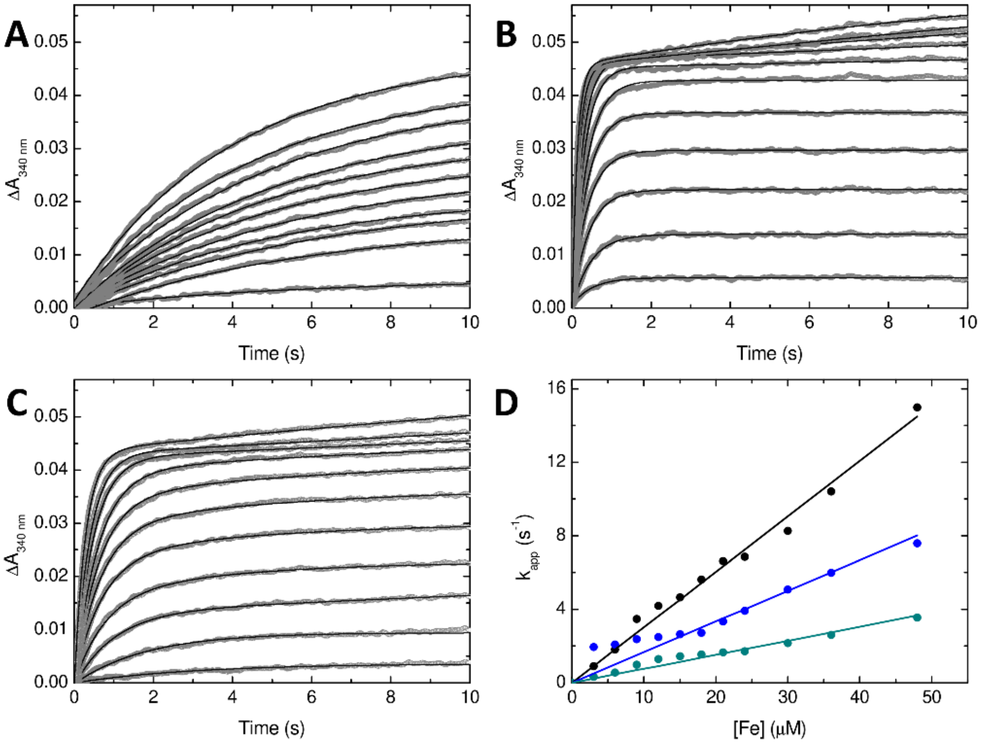

Figure S10. The effect of disruption of the proposed Fe^{2+} uptake route on rapid oxidation activity. The increase in absorbance at 340 nm following aerobic mixing of (**A**) D131A, (**B**) E134A and (**C**) E140A FtMt with Fe^{2+}. Final concentration of protein following mixing was 0.5 μM and of iron ranged from 3-48 μM. Data (grey circles) were fitted to mono- (panel A) or bi- (panels B and C) exponential decay functions (black traces). Panel (**D**) shows the linear dependence of the pseudo first-order rate constants associated with the rapid phase of Fe^{2+} oxidation for E134A (blue) and E140A (dark cyan) on the concentration of Fe^{2+} with the equivalent data for wild-type FtMt for comparison (black). Variant D131A exhibited no rapid phase of Fe^{2+} oxidation. The gradient of each plot is the apparent second-order rate constant (with units of $M^{-1}\ s^{-1}$), see Table 3 of the main paper.

Supporting References

(1) Evans, P. R.; Murshudov, G. N. How good are my data and what is the resolution? *Acta Crystallogr D Biol Crystallogr* **2013**, *69* (7), 1204-1214. DOI: 10.1107/S0907444913000061.

(2) Sievers, F.; Wilm, A.; Dineen, D.; Gibson, T. J.; Karplus, K.; Li, W.; Lopez, R.; McWilliam, H.; Remmert, M.; Soding, J.; et al. Fast, scalable generation of high-quality protein multiple sequence alignments using Clustal Omega. *Mol Syst Biol* **2011**, *7*, 539. DOI: 10.1038/msb.2011.75.

(3) Robert, X.; Gouet, P. Deciphering key features in protein structures with the new ENDscript server. *Nucleic Acids Res* **2014**, *42*, W320-324. DOI: 10.1093/nar/gku316.

(4) Behera, R. K.; Theil, E. C. Moving Fe^{2+} from ferritin ion channels to catalytic OH centers depends on conserved protein cage carboxylates. *Proc Natl Acad Sci U S A* **2014**, *111* (22), 7925-7930. DOI: 10.1073/pnas.1318417111. Tosha, T.; Ng, H. L.; Bhattasali, O.; Alber, T.; Theil, E. C. Moving metal ions through ferritin-protein nanocages from three-fold pores to catalytic sites. *J Am Chem Soc* **2010**, *132* (41), 14562-14569. DOI: 10.1021/ja105583d.
